# Estimating Heat‐Related Exposures and Urban Heat Island Impacts: A Case Study for the 2012 Chicago Heatwave

**DOI:** 10.1029/2021GH000535

**Published:** 2022-01-01

**Authors:** Kaiyu Chen, Andrew J. Newman, Mengjiao Huang, Colton Coon, Lyndsey A. Darrow, Matthew J. Strickland, Heather A. Holmes

**Affiliations:** ^1^ Department of Chemical Engineering University of Utah Salt Lake City UT USA; ^2^ National Center for Atmospheric Research Boulder CO USA; ^3^ School of Public Health University of Nevada Reno NV USA

**Keywords:** urban meteorology, NWP, land surface model, heat stress, Excessive Heat Factor

## Abstract

Accelerated urbanization increases both the frequency and intensity of heatwaves (HW) and urban heat islands (UHIs). An extreme HW event occurred in 2012 summer that caused temperatures of more than 40°C in Chicago, Illinois, USA, which is a highly urbanized city impacted by UHIs. In this study, multiple numerical models, including the High Resolution Land Data Assimilation System (HRLDAS) and Weather Research and Forecasting (WRF) model, were used to simulate the HW and UHI, and their performance was evaluated. In addition, sensitivity testing of three different WRF configurations was done to determine the impact of increasing model complexity in simulating urban meteorology. Model performances were evaluated based on the statistical performance metrics, the application of a multi‐layer urban canopy model (MLUCM) helps WRF to provide the best performance in this study. HW caused rural temperatures to increase by ∼4°C, whereas urban Chicago had lower magnitude increases from the HW (∼2–3°C increases). Nighttime UHI intensity (UHII) ranged from 1.44 to 2.83°C during the study period. Spatiotemporal temperature fields were used to estimate the potential heat‐related exposure and to quantify the Excessive Heat Factor (EHF). The EHF during the HW episode provides a risk map indicating that while urban Chicago had higher heat‐related stress during this event, the rural area also had high risk, especially during nighttime in central Illinois. This study provides a reliable method to estimate spatiotemporal exposures for future studies of heat‐related health impacts.

## Key Points

- Extreme heatwave induced ∼4°C increase in temperature in rural Chicago while increased urban temperature by 2–3°C
- Urban heat island intensity is estimated to be around 1.44–2.38°C
- Excessive Heat Factor is higher than 50°C^2^ in urban Chicago due to heatwave

## 1 Introduction

Along with urbanization, human‐associated changes in global climate led to worldwide increases in heat events, in terms of both frequency and intensity (Min et al., 2019). Heatwaves (HW) have been demonstrated to increase premature mortality from 6,000 to 136,000 worldwide from the 1990s to the 2000s (Liao et al., 2018). Extreme heat events increase not only mortality and morbidity but also have adverse effects on crop yields and ecosystems across the world (Easterling et al., 2000).

Temperature differences between urban (warm) and the surrounding rural areas (cool) can be defined as an Urban Heat Island (UHI), a common consequence of land‐use changes by human activities (D. Li, Liao, et al., 2019). Human activities, main urbanization, are the core reason behind the UHI effect. Increasing population, expansion in urban areas and less vegetation cover account for the increase in UHI strength over recent decades, while the accumulating impervious materials will also intensify UHIs in the future (Sabrin et al., 2020; Singh et al., 2017). Global average daytime UHI during summer results in about 2.6°C–4.7°C higher urban temperatures than rural. As a result, exposure to high temperatures and associated health risks, such as additional heat‐related mortality, are increased in urban populations and vulnerable groups (Tan et al., 2010; Zhang et al., 2010). Consequently, warmer surface/air temperatures in the urban area cause profound effects on humans, inducing adverse impacts on human health and the social ecosystem (Grimm et al., 2008; Heaviside et al., 2017).

Air temperature (ambient temperature) and surface temperature (the temperature at the ground surface) can both be used to calculate temperature differences (∆T) between the urban (Tu) and suburban/rural (Tr) areas as the index to describe UHIs (Lee et al., 2020; Sheng et al., 2017; Zhou et al., 2013). UHIs are primarily evaluated using in‐situ observations of urban and rural air temperatures. Limitations in recording continuous spatiotemporal temperature variations and measurement uncertainties, especially in rural areas, require another approach to assess UHI effects. Remote sensing techniques provide an alternative approach for mapping urban surface thermodynamic properties. However, without consistent definitions of urban and rural sites, uncertainties and inconsistencies in land use classifications can result in difficulty in accurately determining differences between urban and rural temperature (Grimmond, 2006; Hardin et al., 2018). Satellite measurements are widely used in statistical models, such as the Gaussian surface model, to quantify the intensity of UHI which is known as the UHI footprint. But satellite techniques are limited by the expensive cost to deploy the satellite sensors, low temporal resolution, and environmental factors impacting the retrievals (e.g., clouds) (Mirzaei et al., 2010; Streutker, 2003; Yang et al., 2019). A novel method was developed to overcome the arbitrary urban/rural site selections. This approach uses land‐use fractions and sensible heat fluxes to calculate 2‐m air temperature (T_2m_). Details of this method and applications of this approach were introduced by H. Li, Zhou, et al. (2019).

Compared to monitoring or satellite studies, model simulations have the capability of simulating continuous spatiotemporal urban climate variations during UHI events. Several meteorological modeling approaches to estimate UHIs were introduced by Bahi et al. (2020). The Colorado State University Mesoscale Model was successfully applied in simulating UHI events in Tokyo, concluding that anthropogenic heat had stronger effects in winter while the shortwave radiation was stronger in summer (Ichinose et al., 1999). The ENVI‐met model, a non‐public free software, was applied to evaluate microclimates within urban environments and to simulate outdoor air pollutions (Ambrosini et al., 2014). Maleki and Mahdavi (2016) applied this model and found that increasing vegetation and permeable area could reduce air temperature by up to 3 K during a UHI event in Vienna, Austria.

The Weather Research and Forecasting (WRF) model has been widely used for analyzing urban climate, which provides detailed simulations on urban canopy layers, land use categories, building energy parameters and land surface climate zones (Chen et al., 2011). The National Urban Database and Access Portal Tool (NUDAPT) (Glotfelty et al., 2013) and World Urban Database and Access Portal Tool (WUDAPT) (Ching et al., 2018) were developed to provide urban canopy parameters to support the urban canopy models in WRF. Both the Single‐Layer Urban Canopy Model (SLUCM) and Multi‐Level Urban Canopy Model (MLUCM, coupled with Building Energy Parameterization [BEP]) and Building Energy Model (BEM) use NUDAPT and National Land Cover Database (NLCD) (Homer et al., 2020) data to grid urban parameters to the simulation domain. These databases were successfully applied in analyzing urban climate in Vienna (Austria), Hong Kong (China), Houston (U.S.) and many cities that face increasing risks of UHIs (Hammerberg et al., 2018; Wong et al., 2019). With the updated land‐use data set, He et al. (2007) quantified the UHI intensity in China and estimated an increasing trend of ∼0.11°C per decade in spring. Fu and Weng (2018) tested the temperature differences between various land use types and found the UHI intensity of 3.0–3.9 K during nighttime in Atlanta, Georgia.

Chicago has been reported that suffered a lot from extreme HWs, including one event that caused more than 700 deaths in 1995 (Browning et al., 2006) and another HW event in 2012. This paper focuses on the 2012 HW that lasted from July 4 to 7, 2012 due to prolonged dryness and high pressure in the middle Mississippi Valley and lower Great Lakes regions since late June. Given there is no comprehensive study that analyzed this event, this study provides a model simulation investigation for this HW event and offers an evaluation of UHI effects to provide support for further study of heat‐related epidemiology and economic impacts. UHI effects also significantly increase the urban Chicago temperature compared to the surrounding area. High UHI intensity was observed in Chicago from 1988 to 2010, and the UHI phenomena were observed more obviously in spring and summertime (Mbuh et al., 2019; Wu et al., 2019). Chicago's Climate Action Plan was introduced to mitigate Chicago heat events (Coffee et al., 2010), making Chicago an ideal city for analyzing HWs and UHIs. Though many efforts have been implemented to mitigate UHI impacts in Chicago, a deeper understanding of the mechanisms and impacts of UHI are necessary for urban planning and development. To provide datasets for quantifying UHI, this work evaluates different datasets to estimate HW and UHI impacts and provides a method to calculate spatially resolved heat‐related exposure metrics for use in epidemiologic research in urban environments. Three datasets are used in this work, (a) Measurements from ground‐based networks, (b) an offline (uncoupled) column land‐surface model based on 1‐km CONUS meteorological product that relies on land surface modeling, and (c) WRF simulations with sensitivity testing for the boundary layer physics options. Observational data has its limitations as mentioned above, the NCAR model estimates surface temperatures on a 1 km grid without lateral advection, and WRF increases the complexity by including all atmospheric physics, dynamics and building energy parameters. This work evaluates these datasets to recommend an appropriate method to provide the most reliable characterization of extreme heat and UHIs for future epidemiologic studies.

## 2 Methods

An extreme HW was observed in Chicago from 4 to 7 July 2012, where the highest temperature reported was 39.4°C/103F (NWS, 2012a). Simulations from July 1 to 8 are conducted in this study using different model settings to improve model results during the HW. The models and datasets used in this paper are described below in decreasing order of complexity.

### 2.1 WRF Configuration

**Figure 1:**
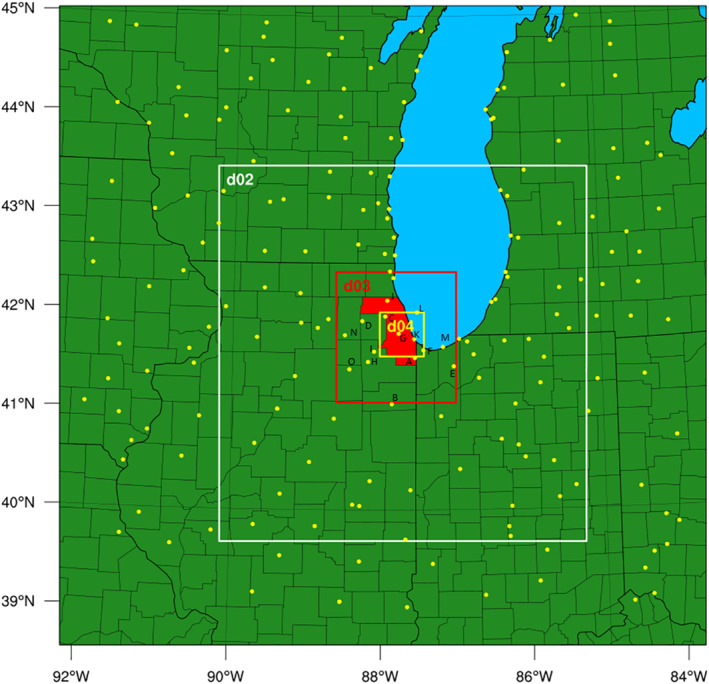
Weather Research and Forecasting domain settings and locations of meteorological observation stations (yellow dots), Cook County that includes urban Chicago is also shown (red shaded area). Observations are obtained from NOAA (https://www7.ncdc.noaa.gov/CDO/cdoselect.cmd).

Numerical simulations were performed with WRF version 3.8 (Skamarock et al., 2008) using a 9 km horizontal resolution outer domain (d01) nested (two‐way nesting) with three finer domains covering the City of Chicago as shown in Figure 1. Nested domains have a ratio of 3 to their outer domain, with the finer‐scale resolutions of 3 km (d02), 1 km (d03) and 333m (d04). A total of 30 vertical layers are used, and six of them are in the lowest 1 km of the boundary layer, with the first layer ∼70 m above ground level. Initial and boundary conditions are obtained from National Centers for Environmental Prediction (NCEP) North American Mesoscale (NAM) 12 km analysis data (NCEP North American Mesoscale (NAM) 12 km Analysis, 2015). NLCD 2011 provided by Multi Resolution Land Characteristics (MRLC, http://www.mrlc.gov) offers land cover monitoring data to assess land‐use variation in this study (Homer et al., 2015). NUDAPT is applied to provide specific morphology of the urban area and support the WRF urban canopy models (Glotfelty et al., 2013).

The first 11 hr before the study period are included in the simulation as model spin‐up (6 model initial spin‐up hours and an additional 5 hr due to the change from UTC to local time zone); results from the model spin‐up were not included in the analysis. Basic settings are shown in Table 1. The Mellor‐Yamada‐Janjic (MYJ) scheme is applied to calculate turbulent kinetic energy (TKE) in this study (Janić, 2001; Kim et al., 2013). To simulate not only urban surface heat, momentum and turbulent kinetic energy but also their vertical distribution in urban canopy layers, BEP (Martilli et al., 2009) is applied to improve simulations in the urban region.

**Table 1:** WRF Configurations for the Three Scenarios

Scenario ID	1	2	3
Scenario name	Default	MLUCM	Nudging
Urban surface physics	N/A	MLUCM_BEP	MLUCM_BEP
Nudging option	N/A	N/A	Nudging on the coarse domain (d01)
Number of urban atmosphere layers	N/A	15	15
PBL scheme^a^	MYJ		
Land surface option	Noah Land‐Surface Model		
Surface layer option	Monin‐Obukhov (Janjic Eta) Similarity scheme		
Longwave/shortwave Radiation option	RRTMG scheme		

*Note*. With increasing model complexity from Scenario 1 to 3.

^a^ Each scenario uses the same settings for the PBL, land surface, surface layer and long/shortwave radiation options.

Observational nudging techniques are developed for data assimilation and are widely used to reduce the uncertainties provided by atmospheric models. This technique is applied to improve global‐regional dynamic consistency in numerical weather modeling (Omrani et al., 2015). Many studies found nudging to provide more accurate temperature, precipitation, moisture fields and reducing system errors from predictions (Glisan et al., 2013). In this study, WRF applies this technique to improve the simulation results for the HW event. Observational nudging is applied in the third scenario for the coarse domain (d01). This is due to the limited availability of observations in the nested domains. Observational nudging is used to assimilate four‐dimensional data including temperature, moisture and wind fields. Upper air temperature and surface observational weather data for nudging are provided by NCEP ADP Global Upper Air Observational Weather Data (NCEP ADP Global Upper Air Observational Weather Data, 2004). Observational nudging was designed to improve model performance in this study. Details on the nudging technique can be found in Reen (2016).

### 2.2 HRLDAS Data Set

The High‐resolution Land Data Assimilation System (HRLDAS) (Chen et al., 2007), an offline (one‐way coupling) system, is used to estimate near‐surface meteorology. It allows for use of historical observed meteorology, in this case, the National Land Data Assimilation System Phase‐2 (Xia et al., 2012), and high‐resolution land cover data (NLCD here), to drive the same land surface models within WRF in a 1‐D column mode, which neglects lateral advection. The low computational cost and strong constraints of using observed meteorology in an offline system make HRLDAS useful for many applications including the examination of UHIs (Monaghan et al., 2014). Here we use the same land‐surface model, Noah land surface model (LSM) was applied in the WRF simulations described above. However, this HRLDAS configuration uses a simpler single layer urban canopy model to simulate near‐surface (2 m) air temperature, and humidity using a 1 km horizontal resolution grid and hourly timestep over CONUS. Results from the offline HRLDAS column model are compared with outputs from our fully coupled WRF simulations to evaluate the benefits and disadvantages of using much simpler methods to simulate the atmospheric conditions in the urban environment during the HW event. It is important to note that HRLDAS uses significantly less computational resources, it takes around one‐eighth of the core hours for the same domain settings compared to WRF.

### 2.3 In Situ Observations

MesoWest (Horel et al., 2002), a cooperative program providing access to real‐time weather observations across the U.S. for weather research, is used in this study. MesoWest provides a web‐based resource to access data that is collected and archived from meteorological networks throughout the U.S. The data accessible from MesoWest provides detailed temperature variations and diurnal changes in urban Chicago during the HW episode. A total of 27 stations have data available for the HW episode within a 40 km radius of downtown Chicago (including one over Lake Michigan), which are chosen to represent the finer scale observations for urban Chicago. Their locations in the third WRF domain (d03) are shown in Figure S10 in Supporting Information S1. Noting that observations are compared with simulations from the finest domain (d04) for those observations located within the domain (d04), model results from the third domain (d03) are used when the station is outside d04. The observation data available from MesoWest have a higher time resolution (∼20 min) compared to the other datasets and WRF simulations (1 hr) in this study. The finer time step helps to better investigate the temperature variations in the urban area.

### 2.4 Model Evaluation

Basic meteorological variables, temperature (T) and relative humidity (RH) 2 m above the surface, and horizontal wind components (wind direction [WD] and wind speed [WS]) 10 m above the surface, are evaluated with observations from the surface data provided by NCDC DS3505 Integrated Surface Data (National Climatic Data Center, https://www.ncdc.noaa.gov/). The station locations are shown in Figure 1. A total of 194 stations in d01 are included in the model evaluation for this study. Fifteen of them are located in urban Chicago and the surrounding suburban/rural area, which are used to evaluate the model performance for urban meteorology. Statistical evaluation metrics including root mean square error (RMSE), mean gross error (GE), mean bias (MB) are calculated and assessed based on the suggested benchmarks given in Emery et al. (2001).

### 2.5 Quantifying UHI Intensity

UHI intensity (UHII) is quantified using a method introduced in a previous study (H. Li, Liao, et al., 2019). In general, a linear function (Equation 1) is formulated to calculate temperature (T) which is then fit to the urban fraction (URB_FRC) as the independent variable. As a result, the slope of the function is used to describe UHII and the intercept is the baseline temperature for vegetation areas (T(vegetation)).

(1) T=URB_FRC×UHII+T(vegetation)

In this study, the urban fraction (URB_FRC) was zero (0%) for both open water and vegetation areas. Only data for vegetation areas are used to calculate UHII when the URB_FRC is zero. URB_FRC is obtained from WPS aggregation using data from NLCD and NUDAPT, indicating the level of urbanization and representing the fraction of impervious areas. The urban categories are classified as low‐intensity residential, high‐intensity residential, and industrial/commercial with urban fractions of 50%, 90%, and 95%, respectively in the NLCD.

### 2.6 Heat‐Related Exposure Assessment

The heatwave‐associated exposure is estimated using the Excess Heat Factor (EHF) which is a metric to quantify the intensity of heat (Borg et al., 2019; Jegasothy et al., 2017). EHF was developed by the Australian Bureau of Meteorology and successfully applied not only in Australia but also in other countries with diverse climates, such as the U.S. (Huang et al., 2021; Varghese, Hansen, Bi, et al., 2019). In general, EHF is estimated with two excess heat indices (EHIs), EHI_sig and EHI_accl, which are calculated as shown in Equations 2 and 3.

(2) EHI_sig=T3−T95

(3) EHI_accl=T3−T30

where T3 and T30 in Equation 3 represent the mean temperature in the previous consecutive 3and 30 days, respectively. T95 refers to the 95th percentile of mean temperature across the previous 10 years (2003–2012). In this study, T3 is calculated based on the temperature from July 4 to 6 to represent the HW impacts. EHI_sig is used to indicate an absolute value for a warm trend during the heat period. A positive value means that local temperatures for the three consecutive days are hotter than in previous years. EHI_accl is the acclimatization index to evaluate the temperature anomalies in the recent past (30 days). Therefore, the EHF can be obtained from Equation 4 as the product of two EHIs. More details related to the EHF can be found in previous studies (Huang et al., 2021; Nairn & Fawcett, 2015)

(4) EHF=EHI_sig×MAX(1,EHI_accl)

Note that 10 years of data are needed for calculating T95, while WRF in this study provides simulation results only for the 2012 summer. We chose to use the Daymet data set (Thornton et al., 2014), which has daily surface weather data on a 1 km grid in North America since 1980, to calculate T95 in this study. To compute T30, we perform a full summer 2012 WRF simulation using configuration 2 (Table 1) as it has the best performance as compared to observations (determined in this study, see Sections 3.1 and 3.2).

## 3 Results and Discussion

### 3.1 Urban Meteorology Model Evaluation

Overall WRF model performance is evaluated using 197 stations within the coarse domain, results can be found in Figures S1 and S2 in Supporting Information S1. To evaluate model performance in the Chicago urban/rural area, hourly temperature from the third domain (d03, which covers Chicago and the surrounding suburban and rural area) are evaluated and the statistical results are shown in Table 2. A total of 15 monitoring stations are used and five of them are in the Chicago urban area. MLUCM and Nudging have notable lower temperature MB and GE than the Default scenario. Significant improvements are found in the urban area (i.e., stations A, C and G) where applications of urban canopy model lower MB in MLUCM and Nudging than Default scenario (MB > 3). Compared to the Nudging evaluation, though very close, MLUCM has slightly lower MB and GE, especially in the urban area except at stations C and L. MLUCM has an average MB and GE of 0.99 and 2.26, respectively. Comparison of statistical metrics in Table 2 reveals that MLUCM has a better performance for temperature simulations in Chicago and the surrounding area compared to the other model configurations. Even though MLUCM has the best performance, exceedances in the benchmarks for the evaluation metrics still exist. Performance metrics improve by increasing the sample size and with longer averaging periods. The suggested benchmarks are more suitable for a simulation over a month while the metrics here are averaged over one week. In addition, this study improves the WRF model performance in the urban area by using MLUCM which has less of an impact in rural areas and uncertainties remain.

**Table 2:** Statistical Analysis of MLUCM Model Performance Using Temperature From 15 Stations

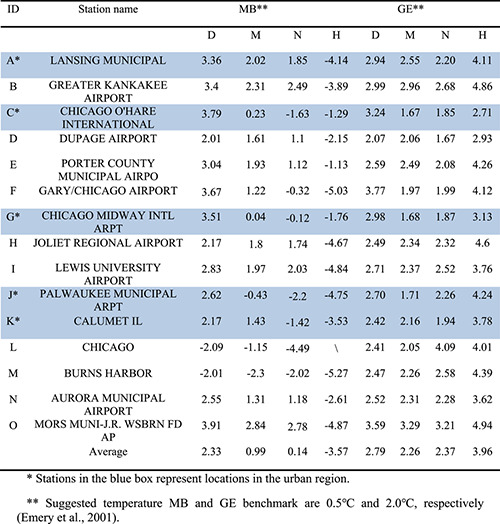

**Figure 2:**
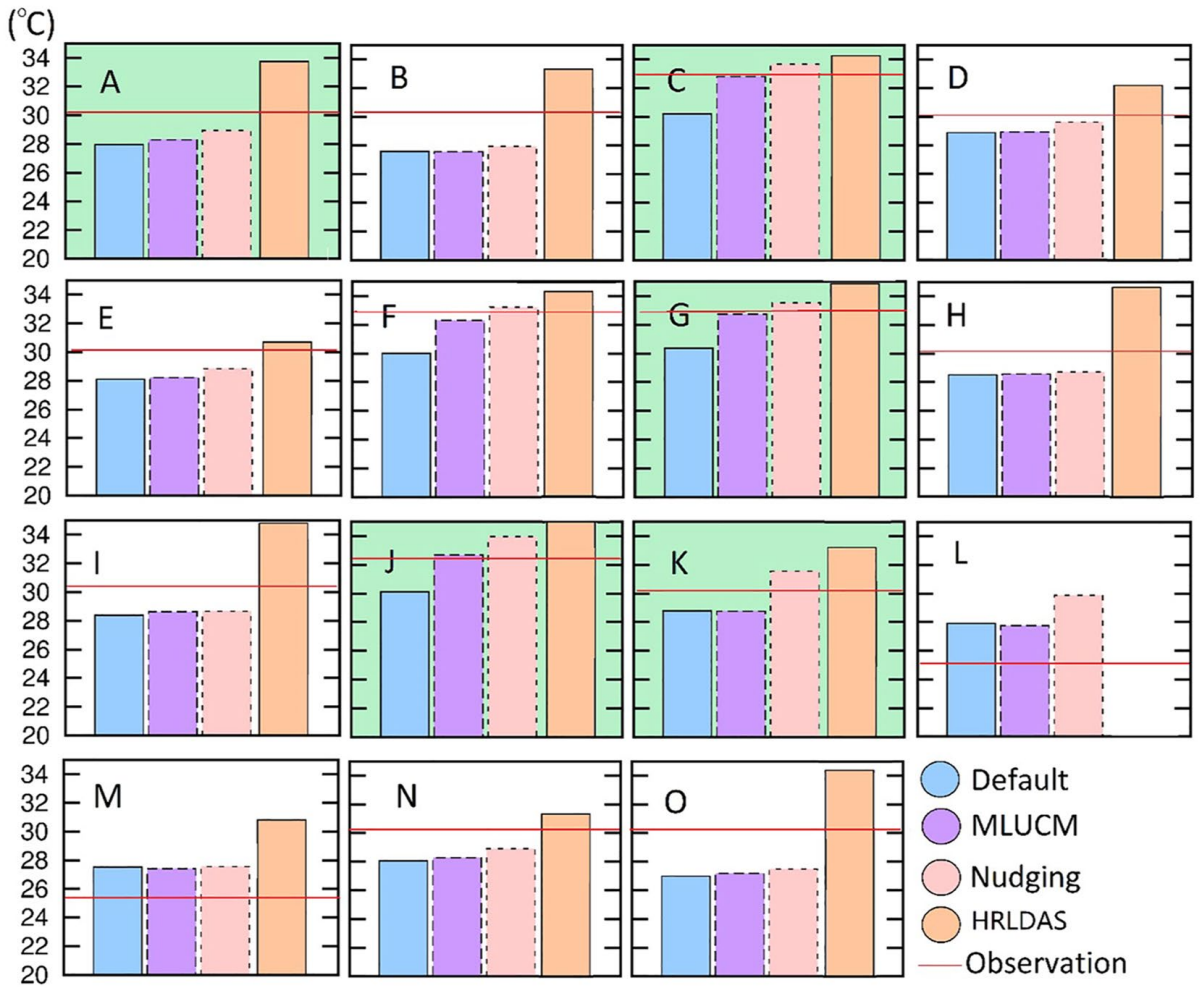
Comparison of daily average (July 1–8) temperature (T) from four model scenarios with observations at 15 meteorological stations near Chicago. Panels with green shading are the stations located in the urban area of Chicago.

Results from HRLDAS are also evaluated (Table 2 and Figure 2). In general, HRLDAS tends to overestimate the temperature, and the statistical metrics reveal that results from HRLDAS have higher biases than WRF (e.g., Monaghan et al., 2014). The statistical metrics also reveal that HRLDAS has slightly better performance in urban areas than suburban/rural areas. HRLDAS calculates the surface temperature by weighting the vegetated surface temperature (non‐urbanization surface) by the urban fraction. Impacts of urban energy transport cause complex variations of the air temperatures in urban regions. The HRLDAS results indicate that the weighted factors help to describe the urban temperature with less bias even though HRLDAS has a higher overestimation in this study compared to WRF. Overall, results from HRLDAS match the spatial pattern but overestimate the temperature.

To visualize the temperature biases further, averaged temperatures for stations and simulations from WRF and HRLDAS are shown in Figure 2. HRLDAS has significant overestimation in Chicago while WRF has less biased simulation results in this study. For WRF, including parameterizations for the urban physical mechanisms (i.e., building energy and urban canopy layer parameterizations) help to simulate higher temperatures than the Default and match better with observations. Meanwhile, MLUCM provides the best model performance in this study. Performance of RH and WS are also shown in Figures S3 and S4 in Supporting Information S1. In general, there are unavoidable biases in each scenario, and results do not always match observations due to complex topography and meteorological conditions in Chicago, especially during an extreme event. Biases also come from the model itself and uncertainties in the initial and boundary conditions. The application of MLUCM provides acceptable simulation results for the Chicago urban area. Nudging technique, though improves the simulation to some extent (such as at station F), it has insignificantly effort in lowering biases and even cause extreme underestimates (i.e., station L). Low overall MB in Nudging (0.14) is mainly due to several underestimations instead of representing better performance. Thus, MLUCM (MB: 0.99, GE: 0.26) is considered to provide the best representation of meteorological variations in urban Chicago and the surrounding area in this study.

These results indicate that WRF successfully estimates the meteorological conditions and urban meteorology during this period in Chicago based on the benchmarks above. The MLUCM improves the model performance significantly, especially in urban areas where heat transfer between different building materials and urban surfaces accounts for variations in the meteorological conditions, which are included in BEP. The nudging technique is only applied in the coarse domain to provide improved initial and boundary conditions for the finer domain in this study. This treatment results in overall higher temperatures than the other two WRF scenarios, especially in urban areas. The HRLDAS model also successfully estimated the spatiotemporal HW variations in Chicago, but with a significant bias. Overestimates in HRLDAS mainly due to it calculates the surface ambient temperature by weighted vegetated surface temperature with the factors derived from urbanization. However, due to the uncertainty of using the SLUCM, HRLDAS provides higher weighted factors, which induce overestimation of surface ambient temperature. WRF conducts full atmospheric dynamics with the multi‐layer urban canopy model, which better represents the atmospheric dynamics in the urban area and provides more reliable simulations. Due to the higher biases than WRF, results from HRLDAS are not included in the analysis for the following sections.

### 3.2 Heatwave and Mesoscale Meteorology

**Figure 3:**
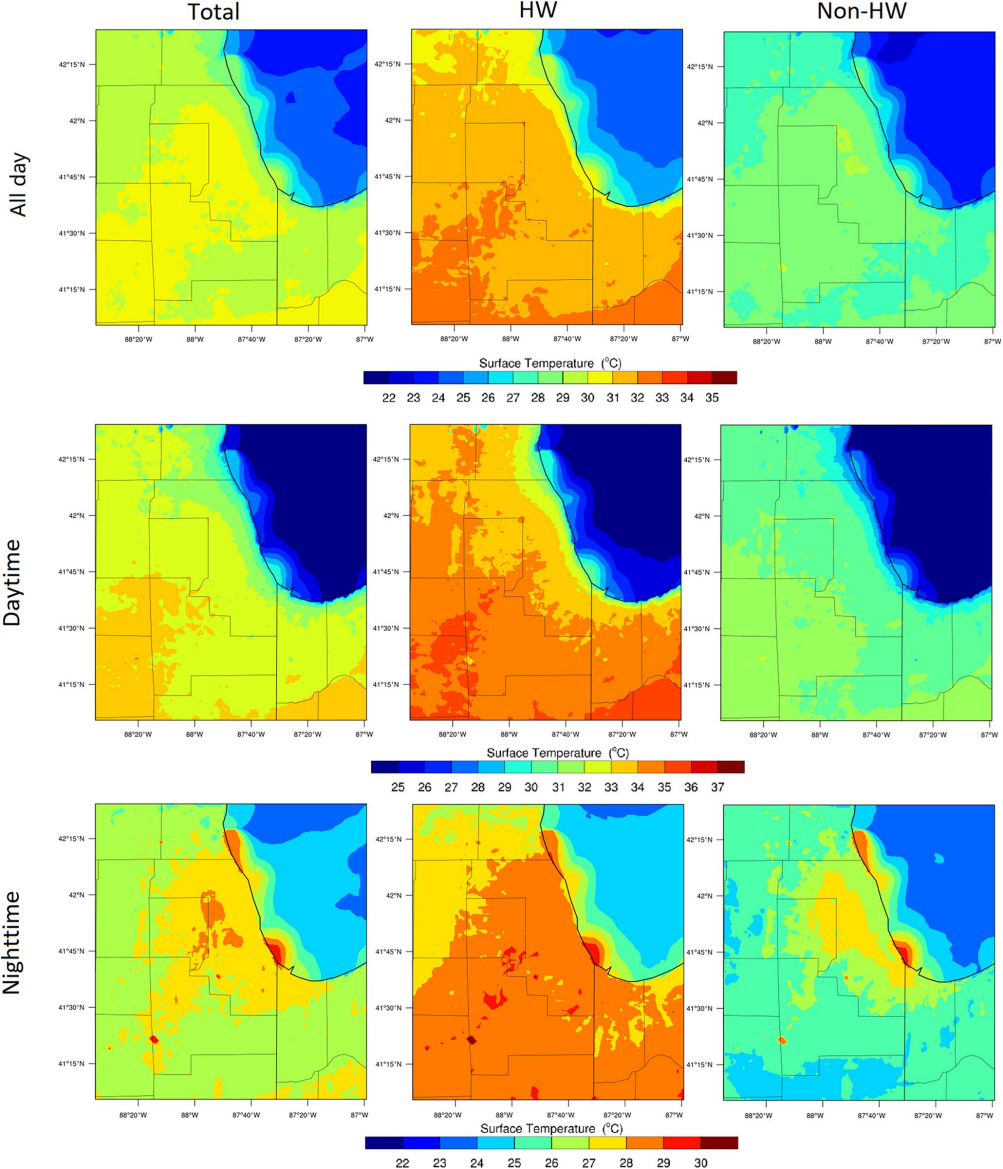
Average temperature in the Chicago region (d03) during the study period (July 1–8, left column), heatwave event (July 4–7, middle column) and non‐heatwave period (July 1–3 and July 8, right column).

To support further heat‐related epidemiology analysis, this simulation provides a detailed analysis of changes of urban meteorology during this HW event. Simulated temperatures from MLUCM during different periods (HW and non‐HW) for the third domain are shown in Figure 3. Overall, the average temperature (during July 1‐8) is ∼30°C in the Chicago urban and suburban areas, which is slightly higher than the surrounding rural area by ∼1°C. It is noted that the rural area in the southwest of the third domain (d03) has similar temperatures as the urban region which is slightly higher than the other rural areas. During the HW event (July 4 to 7), average temperatures in urban Chicago increased to ∼31°C. Concurrently, higher temperatures (32°C) also occur in the south and southwest regions around Orland Park. Compared to the non‐HW period, urban temperatures increase by more than 2°C and rural areas have a significantly higher increase (∼3°C).

Temperature changes during daytime (6:00 a.m.–6:00 p.m.) and nighttime (6:00 p.m.–6:00 a.m.) help describe HW and UHI effects. The average temperature is higher than 33°C during the HW and the highest temperature occurs in north Joliet (41.51°N, 88.08°W) with a maximum of ∼35°C. The average daytime temperature during the HW is ∼4°C higher than the non‐HW period in both urban and rural areas. Urban Chicago has significantly higher nighttime temperatures compared to the surrounding area (by ∼2°C). Compared with urban‐suburban areas, the rural area has higher nighttime temperatures during the HW event. Nighttime rural temperatures increase from ∼25°C to ∼28°C, reaching a maximum of more than 29°C in the region southwest of Chicago.

During the simulation period, urban and suburban areas have notable high nighttime temperatures compared to the rural area while the rural area has slightly higher temperatures during daytime (left column in Figure 3). The nighttime temperature during the HW event is higher in all of Chicago and the rural area to the south (∼28°C). The maximum temperature (>30°C) occurs in Will County (41.45°N, 88.17°W). The HW significantly increases nighttime temperatures by 2°C in the urban region and causes a more than 4°C increase in the rural area. A notable dryness and warm conditions in south Illinois were recorded from May to early July that enhanced hot conditions in this area (NWS, 2012a) As a result, central and south Illinois tended to have higher temperatures.

Wind speeds (Figure S5 in Supporting Information S1) during the daytime are lower in the Chicago urban area (≤2 m·s^−1^) compared to the rural area (∼3 m·s^−1^) during the HW event. Urban Chicago has significantly lower wind speeds compared with the rural area during the daytime. Wind speeds are less than 2 m·s^−1^ in the urban area, causing less hot air to be advected to the urban area. Meanwhile, winds from the north over Lake Michigan introduce slightly cooler air to urban Chicago, resulting in lower urban temperatures compared with the rural area. Wind speeds are less than 1 m·s^−1^ for the non‐HW during the daytime with the minimum wind speed less than 0.5 m·s^−1^ in urban Chicago. Breezes from Lake Michigan (∼1.5 m·s^−1^) slightly affected the temperature in coastal urban Chicago during this period. Relatively cooler air from Lake Michigan reduces the temperature in eastern urban Chicago. No impact from other regions is observed for the non‐HW nighttime based on the wind field results.

Nighttime wind speeds during the HW event are generally less than 1 m·s^−1^ in urban Chicago. At the same time, no significant impact from Lake Michigan occurs. Northwesterly winds transport heat from urban Chicago over the lake surface, contributing to the relatively high nighttime temperatures (>28°C) above Lake Michigan. This was confirmed by checking the NAM input data, where the sea surface temperatures are significantly higher than the surrounding water surface. The high nighttime temperature here is also associated with high outgoing sensible heat flux (Figure S8 in Supporting Information S1). There is a notable nighttime UHI observed during the non‐HW period. Nighttime urban temperatures are higher than 27°C, which are around 3°C higher than in the rural region. Low wind speeds (<0.5 m·s^−1^) prevent advection of heat from the urban area to other areas. Winds from the rural area (∼1 m·s^−1^) slightly reduce temperatures in the suburban area. Results from the coarse domain (Figure S6 in Supporting Information S1) help to explain the high temperatures in rural areas. The maximum HW temperature impacts occur in western Illinois with the average daytime temperature higher than 37°C, which is much higher than the non‐HW period (by ∼4°C). The heat is transported to Chicago by westerly winds during the daytime. No significant impacts from the lake breeze and cooler air from the north are observed at the same time.

Sensible and latent heat fluxes had similar patterns during HW and non‐HW periods as shown in Figures S7 and S8 in Supporting Information S1. Urban Chicago has high daytime sensible heat fluxes especially in the central urban area where the urban fraction is higher than 95% (Figure S9 in Supporting Information S1). The high urban fraction indicates a large amount of impervious surfaces that reduce evaporation in the urban area. Thus, lower latent heat fluxes occur in the urban area. The average sensible heat flux in urban Chicago is ∼330 W/m^2^ during daytime and even has positive sensible heat flux (∼20 W·m^−2^) during nighttime. On the contrary, the rural area has lower sensible heat fluxes (≤100 W·m^−2^) during daytime and negative values (∼−15 W·m^−2^) at night due to the low fraction of urbanization (≤10%).

The HW tends to raise the temperature by ∼4°C in rural areas and the cities surrounding the Chicago metropolitan area during the HW episode. This event resulted in the second‐highest three‐day average temperatures since 1910, with temperatures over 37.7°C (100°F) for three consecutive days (NWS, 2012a). Though it is a short‐term abnormally hot weather event, the HW episode in this study might have also had adverse effects on crop yield and rural ecosystems and needs further investigation (Matiu et al., 2017).

### 3.3 Urban and Rural Heatwave Impacts

Model outputs in a total of 6 selected locations are used (Figure S9 in Supporting Information S1) to analyze meteorological variations during the HW event from the simulation. There are 3 urban sites (Chicago Midway International Airport [MDW, 41.78°N, 87.75°W]), Museum of Contemporary Art Chicago (MCA, 41.89°N, 87.62°W), O’Hare International Airport (O’Hare, 41.98°N, 87.91°W), 2 suburban sites (Downers Grove [DOW, 41.79°N, 88.05°W] and Naperville [NAP, 41.74°N, 88.23°W]) and 1 rural site (Rural, 41.50°N, 88.33°E) located around 80 km southwest of downtown Chicago. The urban fraction represents local urbanization and can also estimate impervious surface coverage. Downtown Chicago and the surrounding urban area have the highest amount of urbanization, with more than 90% impervious area, while the suburban area has ∼50% impervious area. Rural areas have less than 10% impervious areas. Impervious surfaces have high thermal conductivity compared to vegetation. Hourly temperature variations from these locations are shown in Figure S11 in Supporting Information S1. During the HW event, the peak hourly temperature is higher than 38°C. It should be noted that maximum temperatures on July 6 and 7 are higher than 40°C, which is an extremely high temperature that could cause adverse impacts on human health and the urban ecosystem. The rural area has higher temperatures than the suburban area late in the HW period, while temperatures in the urban region are higher than the surrounding area early in the HW period. The rural area has significantly lower nighttime temperatures than suburban and urban nighttime temperatures. The corresponding changes in heat fluxes can be found in Figures S11 and S12 in Supporting Information S1.

**Figure 4:**
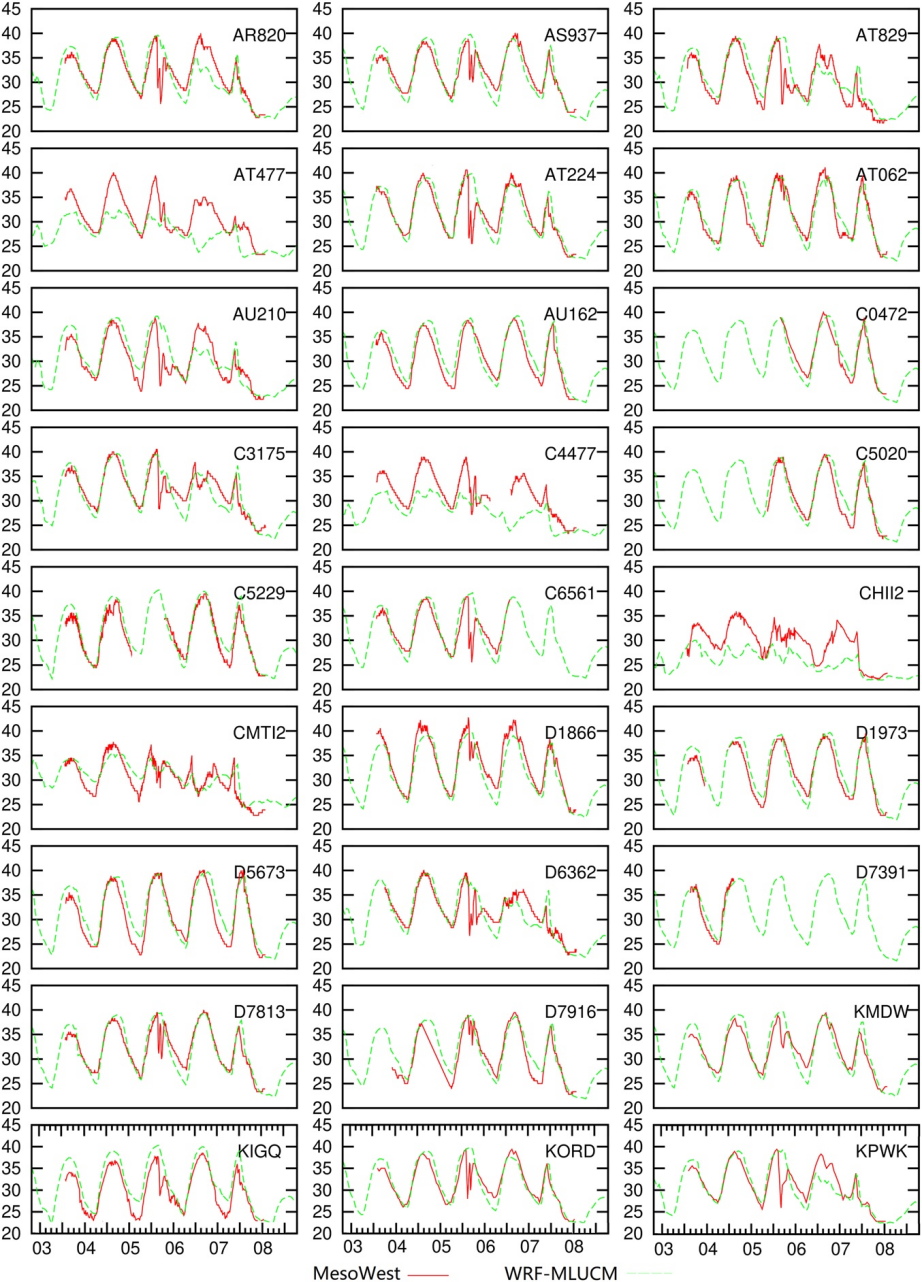
Observations during July 3 to 8 from 27 stations and comparison with WRF‐MLUCM. *X*‐axes represent the dates and the hours. *Y*‐axes show the temperature with units of °C.

Detailed urban temperature changes can be found using meteorological observations. Temperature variations from monitors in urban and suburban regions are illustrated in Figure 4 as well as the comparison with MLUCM. A total of 27 urban/suburban stations reporting to MesoWest provide temperature changes during HW. Noting that part of the data is not available from several stations (e.g., C0472, c5020, and D7391), thus the observation results are only used in discussing temporal temperature variations in urban Chicago. Hourly temperature variations from MLUCM match with the observations in almost all locations except several sites in the coastal area and over Lake Michigan (i.e., CHII2 and C4477). Biases in these stations could be due to uncertainties in representing urban fraction, microscale atmospheric processes and lake effects. On July 5, MLUCM successfully illustrates the daytime maximum and nighttime minimum temperature and most temporal variations. Additionally, there were scattered thunderstorms on 5 July which caused extensive outflows due to the large temperature‐dew point spreads (NWS, 2012b). Such oscillations lasted 3–5 hr before the temperature went back to ∼40°C. However, the MLUCM WRF configuration failed to simulate these storms, which is a common limitation of current numerical weather prediction models. Hazardous convective events, such as short time‐scale heavy precipitation, are hard to simulate and may be underestimated (Chan et al., 2013; Prein et al., 2015).

Daytime maximum temperatures are close to or higher than 40°C which is the highest value in recent years. Locations with a lower urban fraction (e.g., KIGQ and D5673) have a significantly lower nighttime temperature. The drastic fluctuations in afternoon temperature on July 5 are mainly caused by impacts of breezes from Lake Michigan as illustrated in Figure S5 in Supporting Information S1. Significant temperature reductions occur in several coastal stations near downtown Chicago (D6362, C4477 and C3175), highlighting the impacts of lake breezes. Though the overall wind speeds are low, the breezes from Lake Michigan also cause non‐negligible effects on reducing temperatures (by ∼5°C at most) in the coastal urban area. But these mitigating effects are short‐lived and the HW impacts return with temperatures rising to more than 33°C in the evening.

Observations from MesoWest reveal that heat impacts on the coastal urban area are mitigated slightly due to breezes from Lake Michigan but the HW impacts still strongly affect urban nighttime temperatures. Because of the stronger impacts from HWs and fewer temperature alleviations, that is, the absence of lake breezes, rural areas experience more stress from the HW than urban Chicago. Though rural population density is much lower than urban/suburban regions, increased heat stress in the rural area increases the risk of heat‐associated issues. Particularly, because people living in rural areas have less protective support (such as medical resources and social support), they may have less capability to overcome the impacts from extreme heat events (Williams et al., 2013).

### 3.4 Urban Heat Island

**Figure 5:**
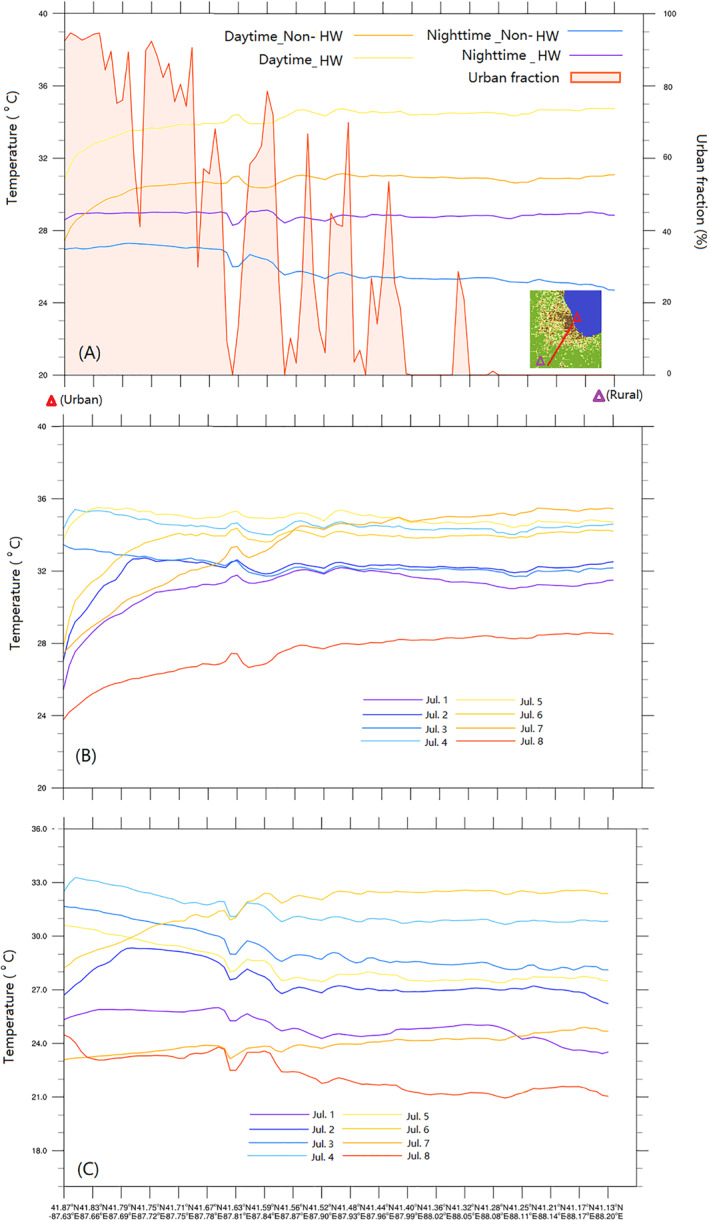
(a) Temperature variations along an urban to rural cross‐section during the day/nighttime for heatwave (HW)/non‐HW periods. (b and c) show the average day/nighttime temperature for each day, respectively. The *x*‐axis represents the location from urban to rural shown in the map subset in (a).

Spatial temperature variations from the urban to the rural areas are shown in Figure 5 based on different periods (HW and non‐HW). In general, urban and rural daytime temperatures increased by ∼3.5°C during the HW episode. However, HW tended to lead to higher nighttime temperature increases in the rural (∼4°C) areas compared to urban (∼1.5°C). Even though the HW results in a high rural temperature, the UHI effects can be observed in the non‐HW period and for some of the HW period. This illustrates the complex interactions between urban (UHI) and mesoscale (HW) meteorological processes that lead to heterogeneous spatiotemporal temperature variations. Where during the non‐HW episode, with fewer impervious surfaces in the low urban fraction area, the rural surface temperature tends to significantly reduce during nighttime. Sharp nighttime temperature reductions occur at the Cook County Forest Preserve (41.63°N, 87.78°W) where vegetation cover predominates (0% urban fraction), and daytime temperature at the same location has notable increases. Corresponding to high temperature in the low urbanized area, sensible heat flux is higher while the latent heat flux and moisture flux are lower in the urban area. Nighttime sensible heat flux decreases below zero in rural and forest preserve areas that indicate the vegetation surface absorbs more heat and reduces the air temperature (Figure S13 in Supporting Information S1).

In Figure 5, panels B and C indicate the daily variations during daytime and nighttime, respectively. Maximum HW impacts during daytime in the urban area occur early in the HW event (July 4 and 5) with an increased temperature of ∼5°C while the temperature in the rural area has a maximum increase late in the HW event (July 7). Decreasing trends from urban to rural (UHI effects) are observed in most nighttime periods except later in the HW event, where temperatures in the rural area are higher than the urban temperatures by a maximum of 2°C. In general, the HW has stronger impacts in the rural areas than in urban Chicago in this study.

**Figure 6:**
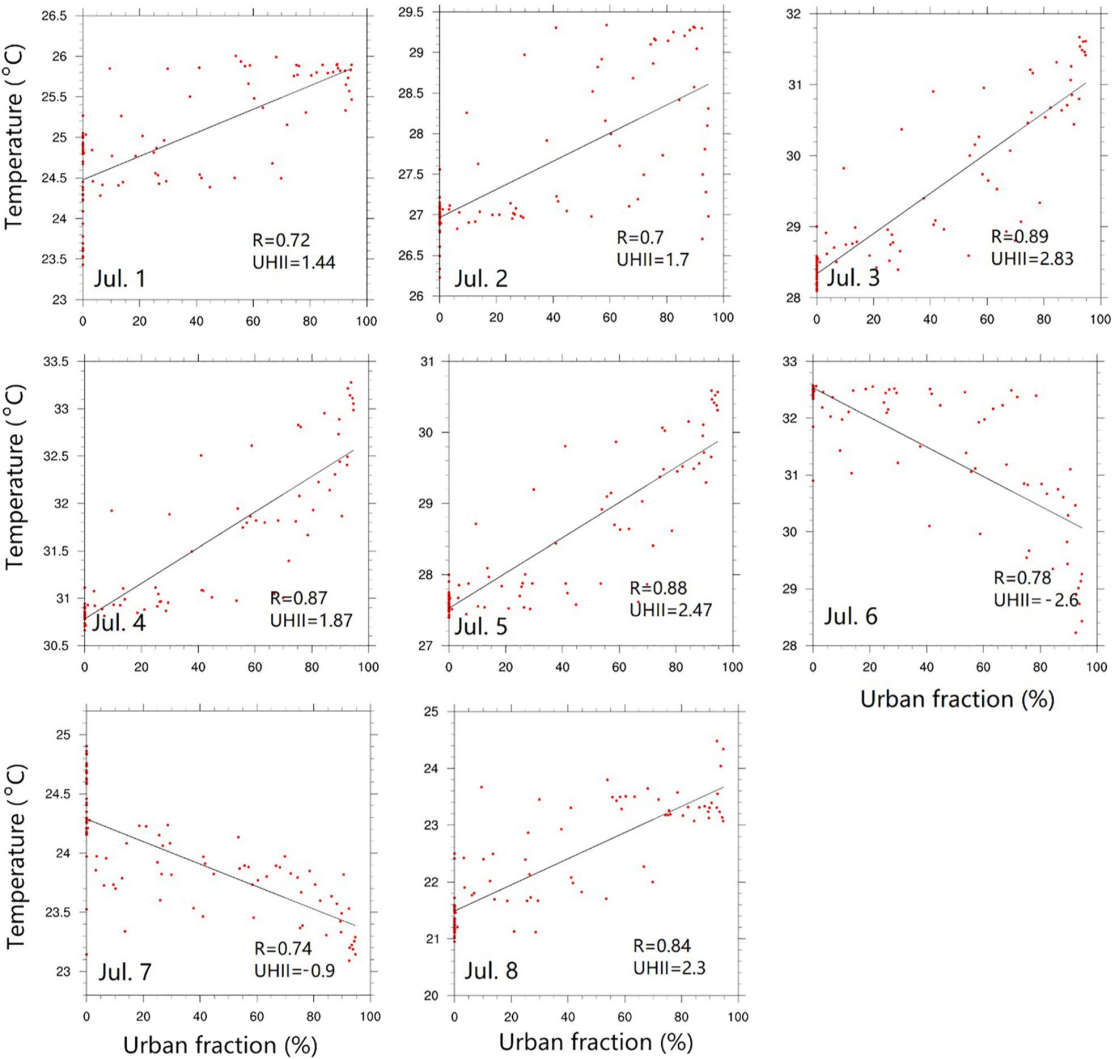
Relationship between nighttime surface temperature and the urban fraction across urban to rural areas (same cross‐section as panel (a) in Figure 5.) Urban Heat Island intensity (UHII) units are °C. *R*‐value for confidences >99%.

UHII during nighttime is quantified (Figure 6), the mean nighttime temperature increases with the increase in urban fraction during non‐HW and early HW periods, which indicates positive relationships between UHII and urbanization. *R* values are higher than 0.7 (*P* < 0.01) on each day, representing strong relationships between them. The maximum UHII is 2.83°C on July 3 while the minimum is −2.6°C on July 6 due to increasing temperature in the rural area caused by the HW. Another negative relationship also occurs late in the HW event with UHII of −0.09°C. The lake breezes from Lake Michigan cause lower temperatures in the higher urban fraction area and mitigated the UHII in the coastal high urbanization areas. Concurrently, strong HW impacts in the rural area increase nighttime rural temperatures and cause negative UHII and urbanization relationships. Both spatial and cross‐section analyses from urban to rural areas indicate that the strongest UHII occurred in central urban Chicago, where the intensity is 1.44–2.83°C.

Long‐term (annual or longer) simulations could be conducted to characterize the overall UHII in urban Chicago. In future work, a long‐term estimation of UHII to support the assessment of associated effects on residents who live in urban/rural Chicago would be beneficial. With reliable UHII estimates, people could evaluate the risk of heat exposure while considering areas to live in. The impacts of increasing impervious surfaces and decreasing vegetation have prolonged effects on urban climate such as higher temperatures, less conducive conditions for advection heat and pollutants (i.e., winds) and lower relative humidity due to less evapotranspiration. Such changes in urban meteorological conditions also deteriorate urban air quality, raise atmospheric pollutant concentrations and extend pollution episodes (Steeneveld et al., 2018).

### 3.5 Heat‐Related Health Risk

**Figure 7:**
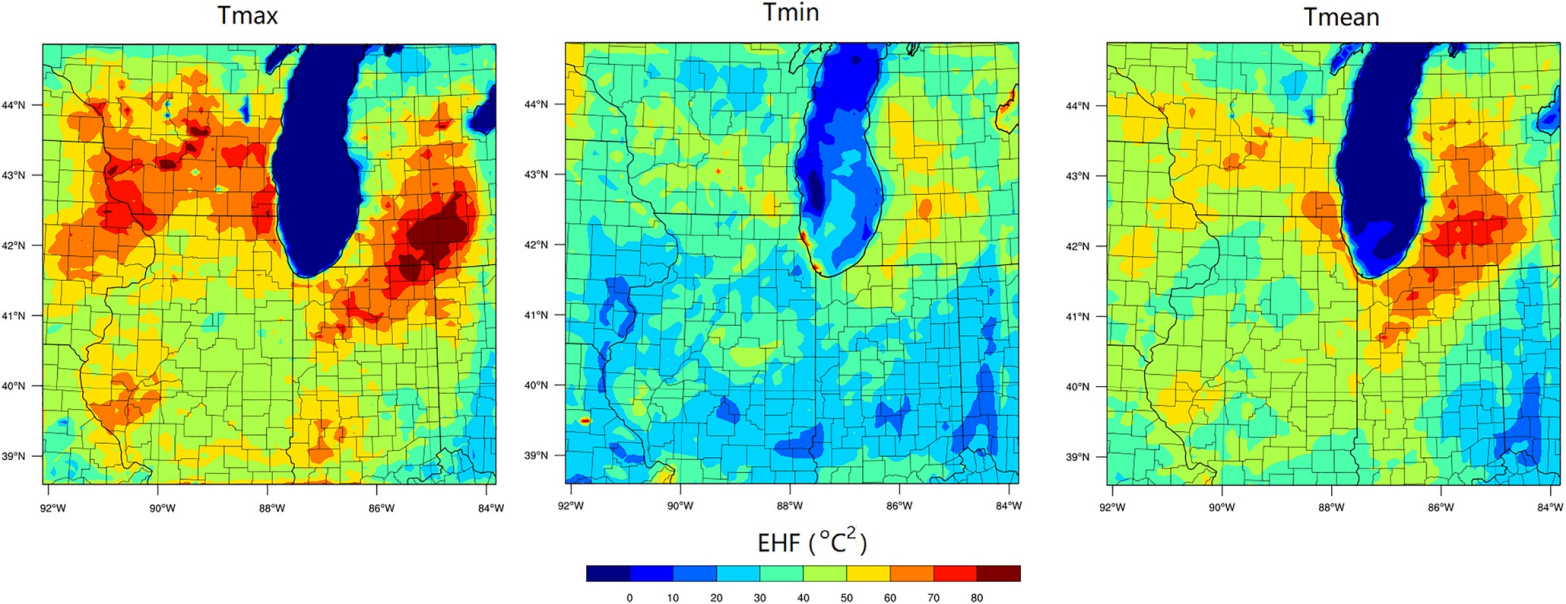
Excessive Heat Factor (EHF) during the 2012 Heatwave. Tmax, Tmin, and Tmean represent the EHF calculated based on daily maximum, minimum, and average temperatures.

To aid in evaluating the potential HW impacts on human health, EHFs are calculated on the largest domain (d01) in this study, shown in Figure 7. Note that the model evaluation results for this domain are included in Supporting Information S1 and the results are shown in Figures S1 and S2. Because the HW had varied diurnal impacts on temperatures, Tmax, Tmin and Tmean are used to illustrate the heat stress based on the daytime, nighttime and the overall average temperatures. In this case, temperatures from July 4‐6 are used to represent the HW T_3_ in the EHF estimation. When considering EHF based on a daily mean temperature (Tmean), urban Chicago has a higher potential health risk compared with the suburban and rural areas during the HW episode. Noting that there is no benchmark directly used to quantify EHF impacts on human health, but several previous studies have linked EHF with potential health endpoints. For example, EHF higher than 2°C^2^ was classified as high severity heatwave and was associated with an increase in work‐related injuries and illnesses (Varghese, Hansen, Bi, et al., 2019). Positive EHF is associated with an increase in emergency department visits (Jian et al., 2015). The EHF is more than 50°C^2^ in the urban area and reaches more than 60°C^2^ in the coastal area, which is more than 20°C^2^ higher than in the rural areas. Interestingly, the heat effects during the daytime (based on Tmax) indicate higher risks in the urban area as well as suburban and rural regions. The HW also causes significant heat stress during nighttime (based on Tmin) in rural areas. The nighttime EHF is more than 30°C^2^ in rural regions in central Illinois and southern Wisconsin with a maximum of more than 40°C^2^. It should be noted that even if EHF is low in urban Chicago during nighttime, the value remains at ∼30°C^2^ in the coastal region.

Outside of Chicago, there are several regions with high EHF estimates in Figure 7. Southern Wisconsin, southern Michigan and northern Indiana have a high EHF during the daytime, reaching more than 80°C^2^. EHF is higher than 60°C^2^ in southern Michigan during nighttime, which is an extremely high value and almost equal to the daytime EHF. Results indicate that the HW not only affects urban Chicago but also has wider impacts on the surrounding states (both urban and rural areas) where the temperatures are normally at a low level (T95 in Figure S14 in Supporting Information S1).

Potential heat‐related impacts have profound adverse effects on human health and socioeconomic impacts. Abnormally high temperatures are associated with acute respiratory and cardiovascular diseases especially in vulnerable populations such as pregnant women and the elderly (Goldie et al., 2018). Meanwhile, rural residents that are used to relatively low summertime temperatures are likely to have less resistance to abnormally high temperatures. The population density is provided in Figure S15 in Supporting Information S1 to illustrate the population exposure to heat‐related risk. As indicated in this study, the HW is associated with an increased temperature in Chicago and the surrounding regions (an increase of ∼4°C in this episode), it causes a daytime EHF higher than 50°C^2^ in the urban area and 40°C^2^ in the rural area. The rural area (low urban fraction area as shown in Figure S9 in Supporting Information S1) also has a high EHF at night (Tmin). Indicating that the increased heat stress associated with acute, synoptically driven HW events does not always correlate with urban fraction. Therefore, heat‐related exposures for spatially resolved human health assessments must consider both the microscale urban impacts (i.e., UHI) and synoptic and mesoscale meteorology (i.e., HW).

A further effort could apply the approach for estimating heat‐related exposures from this study to evaluate the associated health impacts using the nighttime EHF versus daytime EHF. EHF has been widely applied to quantify heat exposure and health impacts from HWs, such as heat‐related morbidity (renal, mental, respiratory and heart‐related illnesses) and mortality (Varghese, Hansen, Nitschke, et al., 2019; Wondmagegn et al., 2021). High daytime EHF in the urban area may cause health problems especially for those who work outdoors. As a consequence, billions of dollars are spent to avoid heat‐related mortality, morbidity and electricity consumption from air cooling systems (McDonald et al., 2020). Results from this study can also be used in future work to evaluate the economic loss during this event.

## 4 Conclusions

This study applied three different WRF configurations and HRLDAS to evaluate model performance in simulating urban meteorology during an extreme heat event in Chicago. Results indicate slightly better model performance when using the MLUCM in WRF. Given the high computational cost and less improvement in providing high‐quality model results when using Nudging, results from the MLUCM scenario without Nudging were selected to analyze the HW and UHI in this study. HRLDAS, though costs less computational resource, provides lower accurate simulation compared to WRF.

With reliable simulations from the MLUCM, the HW and UHI were comprehensively analyzed. The HW during July 4–7 caused notably higher average rural daytime temperatures with a maximum of ∼36°C that were much higher (∼4°C higher) than the non‐HW period. Daytime temperatures in the urban area also increased during the HW period, by ∼3°C. The lake breeze slightly reduced the urban temperature, and low wind speeds during the HW caused high urban nighttime temperatures. UHI effects were observed during nighttime and a strong positive linear relationship (*R* > 0.7, *P* < 0.01) between temperature and urban fraction was found. The UHI was evaluated, and its intensity was quantified. Overall, the nighttime Chicago UHII was around 1.4–2.83°C. The maximum intensity occurred on July 3. The UHI effect was mitigated in the late HW event where rural temperatures were higher than urban temperatures, resulting in a negative relationship between temperature and urban fraction. While these results for specific to Chicago, where a lake breeze is presented, the findings illustrate the importance of considering all spatiotemporal atmospheric scales and their interactions when estimating heat stress.

Considering prolonged HW and intensified UHI effects, impacts of increasing temperature should be noted. Results from this study support further applications in using MLUCM in WRF to investigate urban heat and temperature variations. Reliable short/long‐term simulations help to assess potential effects on human health in epidemiological studies, especially in the urban area where population density is high. Simulations of urban meteorology also provide information for better managing the built environment, urban structures and improving urban ecosystems, such as reducing urban impervious surfaces and increasing vegetation to mitigate UHI effects. With estimated HW and UHI impacts, associated economic losses could also be investigated. This type of investigation is crucial in managing urban development.

## Conflict of Interest

The authors declare no conflicts of interest relevant to this study.

## Supporting information

Supporting Information S1

## Data Availability

We thank NCAR research data archive providing initial and observation nudging data for WRF simulation in this study, data is findable and accessible via https://rda.ucar.edu/datasets/ds351.0/ and https://rda.ucar.edu/datasets/ds609.0/. Historical temperature data set for health analysis was obtained from Daymet which is available via: https://daac.ornl.gov/cgi-bin/dsviewer.pl?ds_id=1840. Observation data from National Climate Data Center's historical information was used to validate model performance in this study, data set is available via http://www1.ncdc.noaa.gov/pub/data/noaa/. MesoWest data set is provided by the University of Utah which is available from https://mesowest.utah.edu/. WPS/WRF and HRLDAS model source codes and associated input datasets are available via https://www2.mmm.ucar.edu/wrf/users/download/get_source.html and https://ral.ucar.edu/solutions/products/high-resolution-land-data-assimilation-system-hrldas.
