# Supplementary material for: Estimating Heat‐Related Exposures and Urban Heat Island Impacts: A Case Study for the 2012 Chicago Heatwave
Source: Geohealth. 2022 Jan 1;6(1):e2021GH000535. doi: 10.1029/2021GH000535 (PMC8772392; doi:10.1029/2021GH000535)
Supplement: Supplementary file 1 — Supporting Information S1 [file GH2-6-e2021GH000535-s001.pdf]

GeoHealth

Supporting Information

Estimating heat-related exposures and urban heat island impacts: A case study for the 2012 Chicago heatwave

Kaiyu Chen[1], , Andrew J. Newman[2], Mengjiao Huang[3], Colton Coon[1], Lyndsey A. Darrow[3], Matthew J. Strickland[3], and Heather A. Holmes[1]

[1] Department of Chemical Engineering, University of Utah, Salt Lake City, UT, USA

[2] National Center for Atmospheric Research, Boulder CO, USA

[3] School of Public Health, University of Nevada, Reno, NV, USA

Corresponding author: Kaiyu Chen (ky.chen@utah.edu)

Number of pages: 17

Number of figures: 15

Number of tables: 1

Simulations from WRF on the coarse domain are evaluated. Simulated T, RH, WD and WS are compared with observed data from 194 monitoring stations in d01 (9 km resolution) and the results are shown in Figure S1. In general, T from each scenario matches well with observations during the study period. It should be noted that using MLUCM (green crosses) tends to slightly overestimate temperature for a few stations at lower temperatures (~22°C). While simulations with nudging (Nudging) have several significant underestimations when temperatures are around 24-29°C. Results from the Default scenario also match well with observations, in general, with slight overestimations. Each of the three WRF model configurations have several notable overestimations of RH and WS.

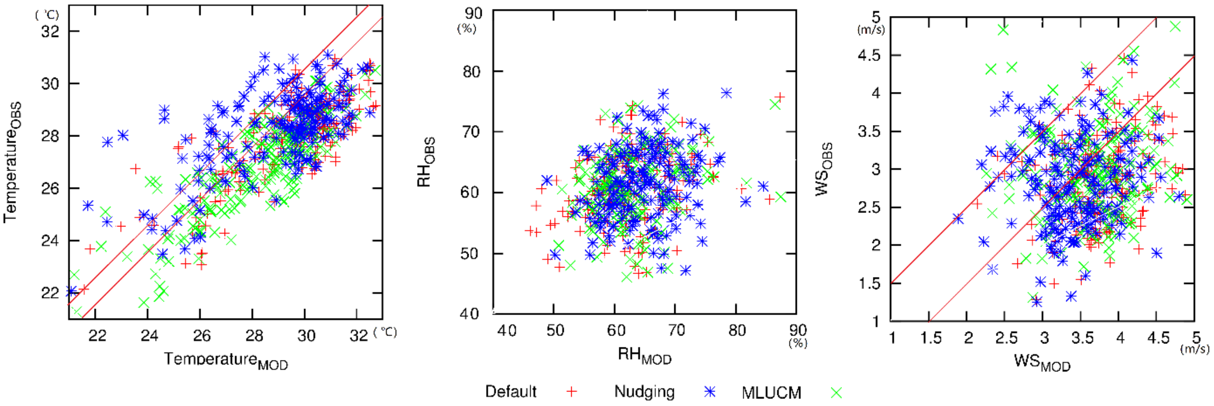

Figure S1. Comparison between model simulation (MOD) and observation data (OBS) for temperature, relative humidity (RH) and wind speed (WS). Red, blue and green dots in figure represent the model results from Default, Nudging and MLUCM, respectively. A total of 194 monitoring stations are included in d01. Red lines in left and right panels indicate the statistical benchmark value for temperature and wind speed.

Statistical analysis of the meteorological model performance for all stations can be found in Figure S2. Temperature GE is 2.41, 2.24 and 2.28 for Default, MLUCM and Nudging,

respectively. And the MB are 1.91, 1.41 and 0.67. Though the nudging reduces MB significantly (by 1.61), it has higher RMSE (2.91) and GE (2.28). Besides, results of WS/WD and RH also indicate that applying MLUCM and Nudging both improve the model accuracy compared to Default, and MLUCM performs slightly better based on the lower RMSE and GE while Nudging has smaller MB for WS/WD. The overall performance of the meteorological simulations are shown in Table S1. Though some of them exceed the suggested criteria (listed in Table S1), both the MLUCM and Nudging scenarios simulate overall acceptable results for this study.

Table S1. WRF model performance (d01, 194 monitoring stations) for meteroglogical variables (MB, mean bias; GE, gross error; and RMSE, root mean square error).

	Statistics	Default	MLUCM	Nudging	Benchmark
T (°C)	MB	1.91	1.41	0.67	≤±0.5
	RMSE	3	2.88	2.91	
	GE	2.41	2.24	2.28	≤2
WS(m/s)	MB	0.89	0.68	0.6	≤±0.5
	RMSE	2.25	1.92	2.19	≤2
	GE	1.75	1.48	1.67	≤2
WD(^{o})	MB	8.07	1.92	-1.74	≤±10
	RMSE	58.81	58.82	63.35	
	GE	42.74	42.01	47.17	≤30
RH(%)	MB	1.56	1.5	4.04	
	RMSE	0.12	0.13	0.22	
	GE	0.1	0.1	0.18	

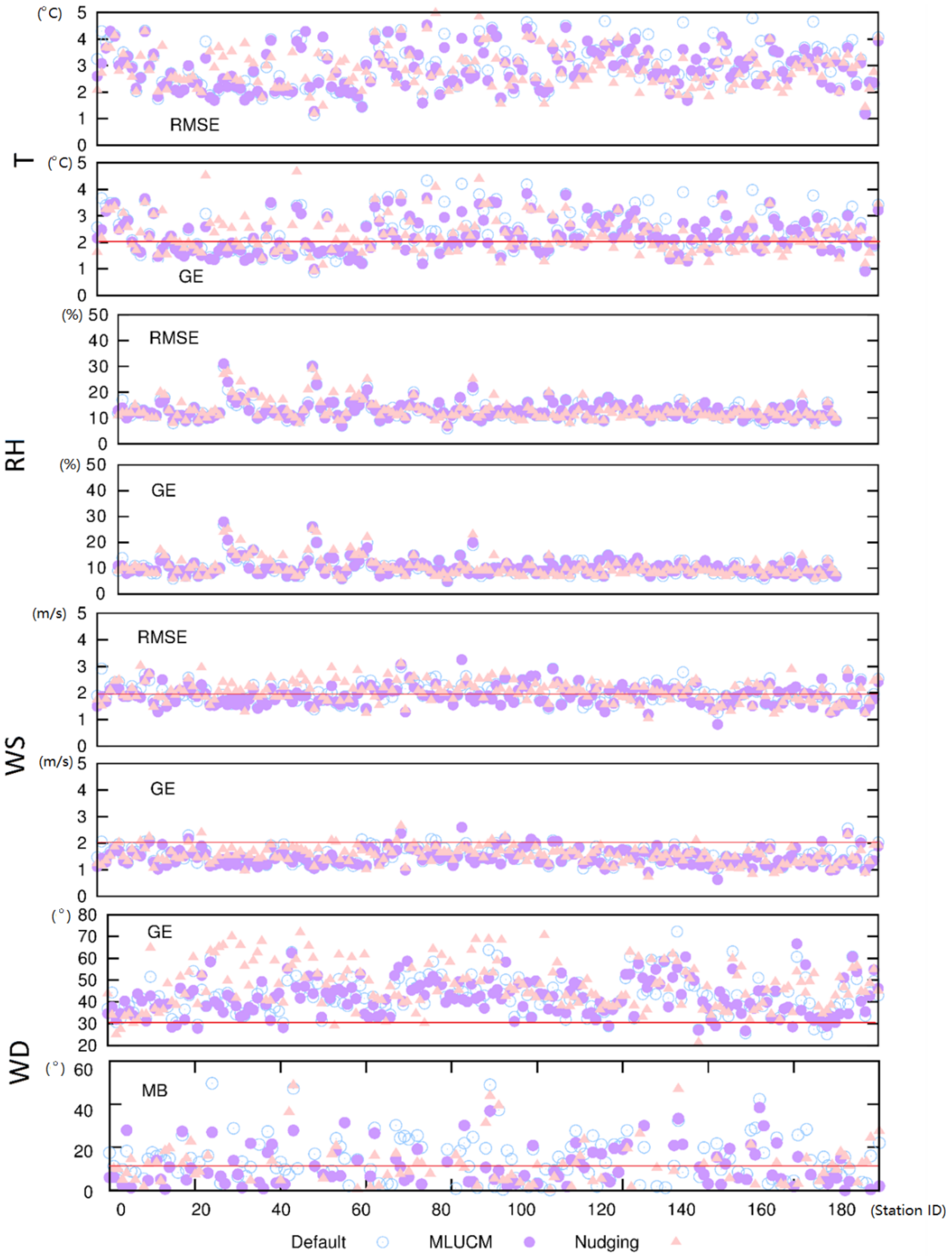

Figure S2. Overall statistical index (RMSE, GE and MB) for temperature, relative humidity and wind speed for each monitoring station (Y-axis). Blue circles, purple dots and pink triangles

represent results from Default, MLUCM and Nudging scenarios, respectively. Red lines in each panel represent the benchmarks.

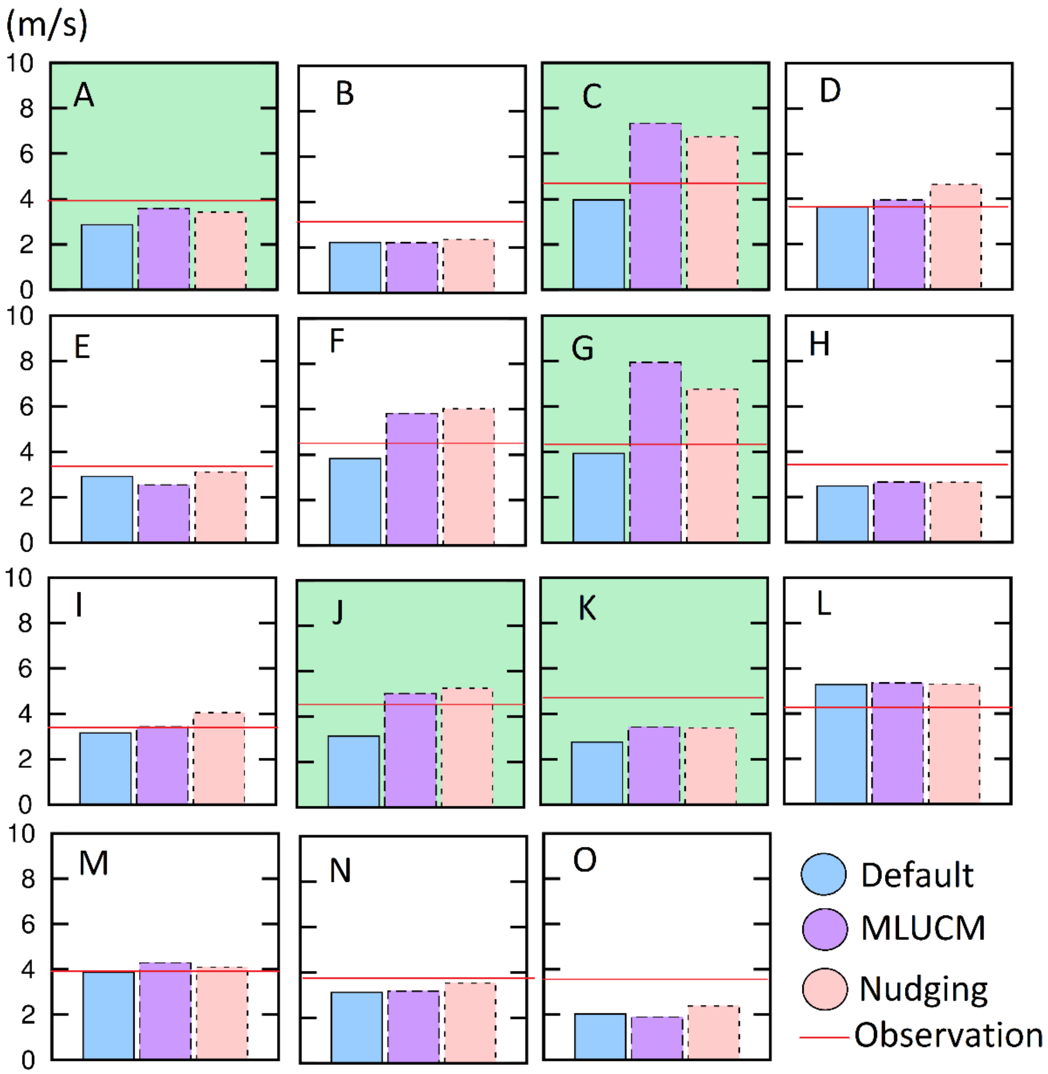

Figure S3. WRF model performance for wind speed (WS) simulations and comparison with observations at 15 stations in Chicago and the surrounding area (d03).

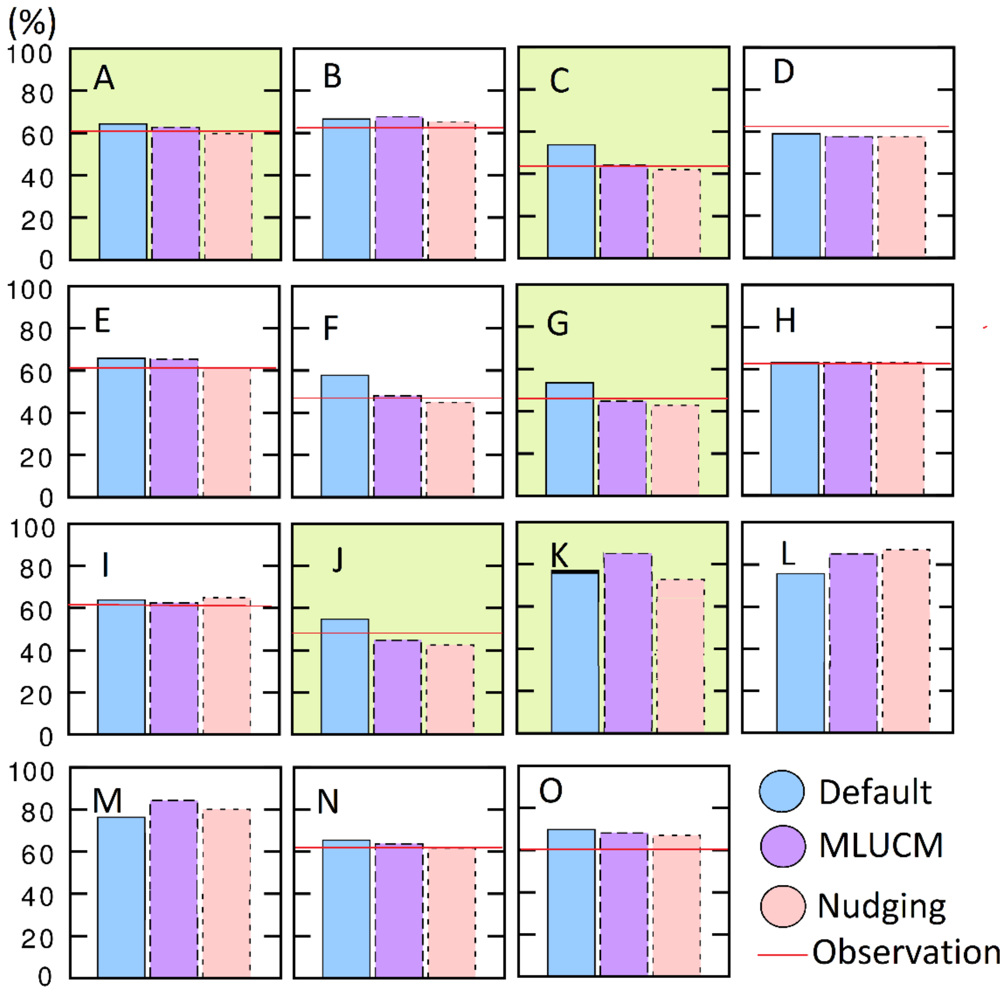

Figure S4. WRF model performances for relative humidity (RH) simulations and comparison with observations at 13 stations in Chicago and the surrounding area (d03). *Note: Station K, L and M has no recorded RH data.*

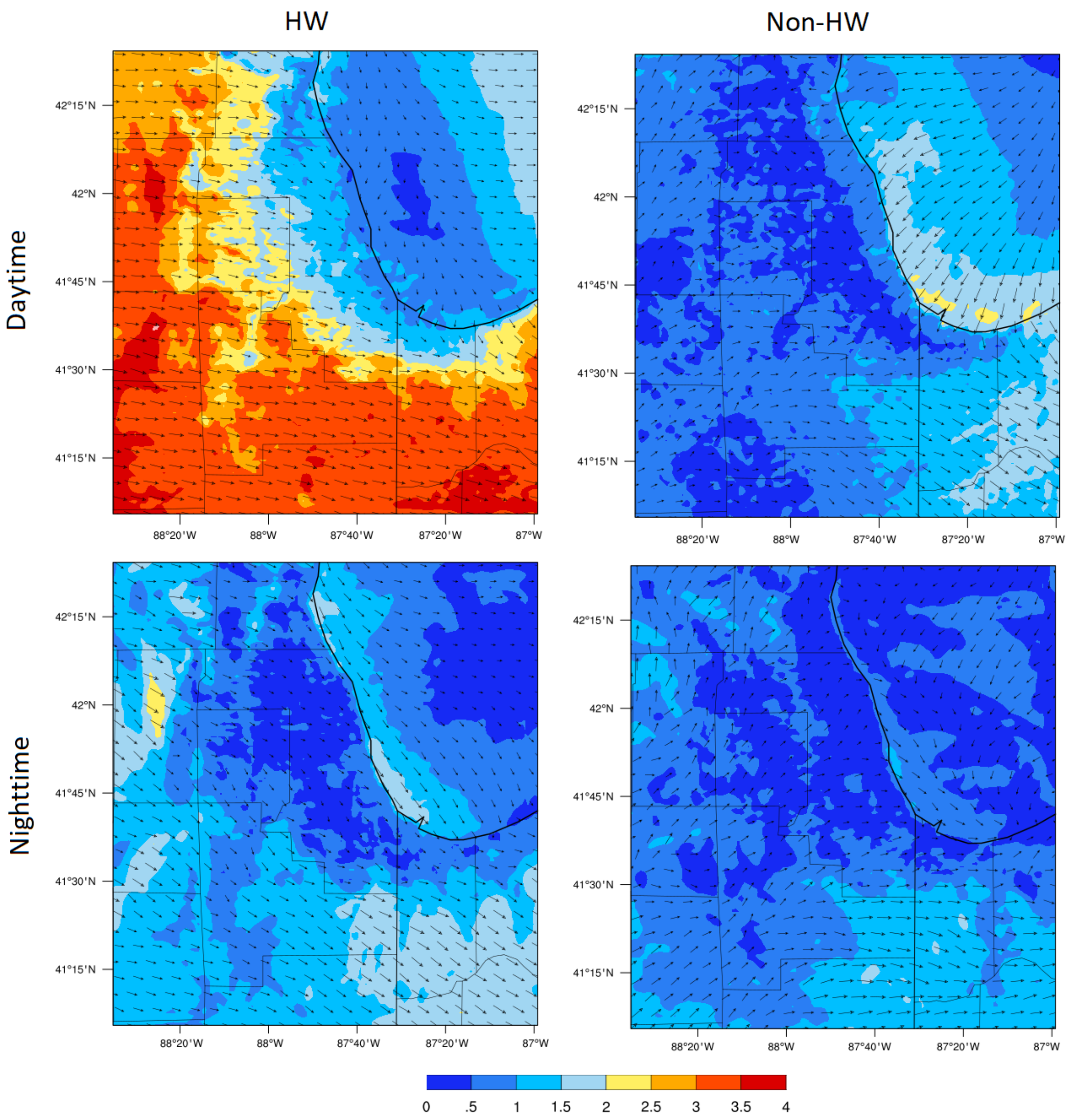

Figure S5. Wind fields for daytime (top) and nighttime (bottom) during UHI and non-UHI periods. The color axis in each plot represents wind speed intensity, units are m s^{-1}. Arrows represent wind direction.

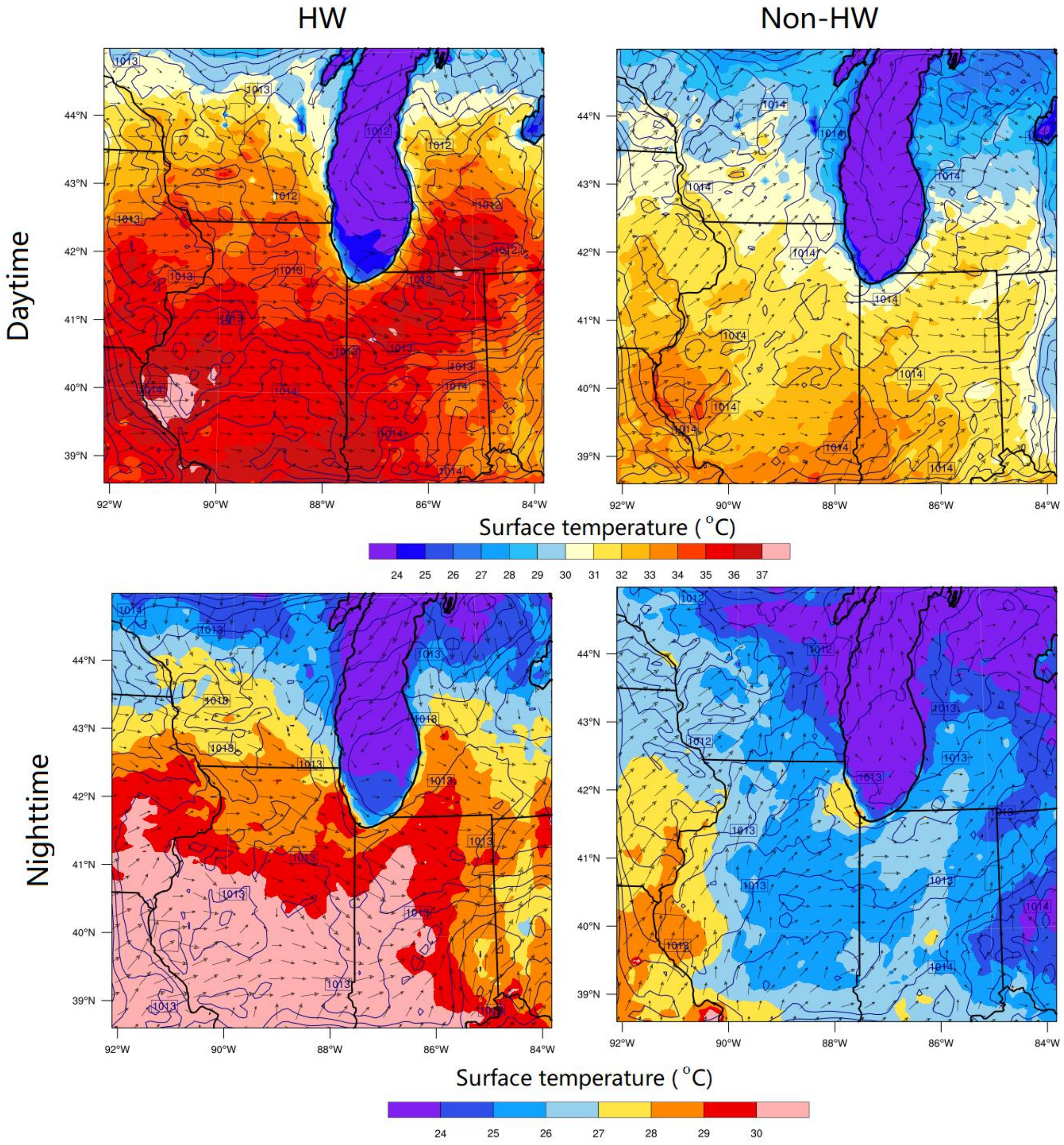

Figure S6. Wind direction (arrows), surface temperature (color axis, units are °C) and sea level pressure (lines, units are hPa) for the coarse domain (d01). Top panel represents daytime average and bottom is nighttime average. Left column is the average for the HW event and right column is the average for non-HW periods.

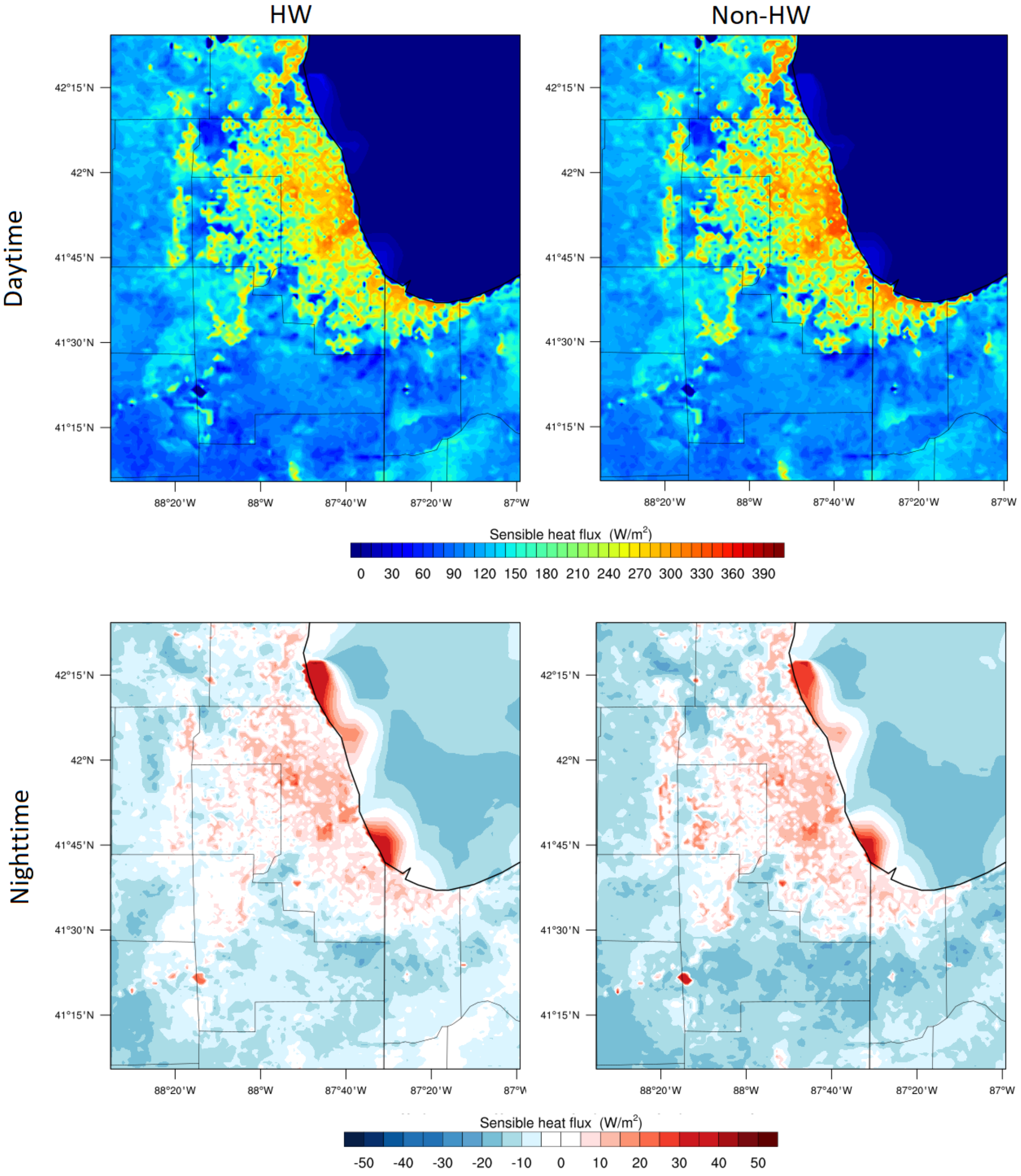

Figure S7. Sensible heat flux (W m^{-2}) for the third domain (d03). Top panel represents daytime average and bottom is nighttime average. Left column is the average for the HW event and right column is the average for non-HW periods.

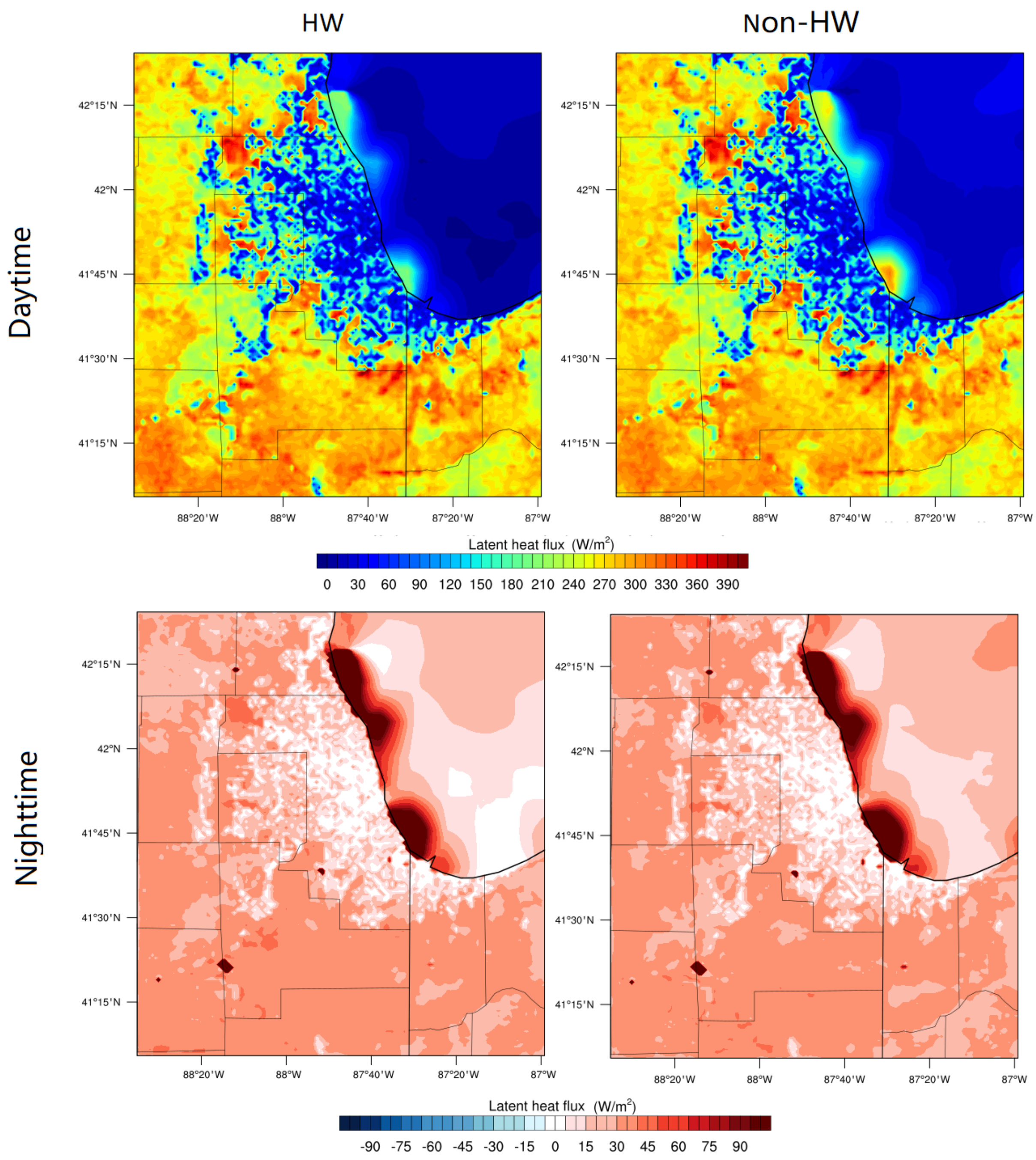

Figure S8. Similar to Figure S7 but for latent heat flux (W m^{-2}).

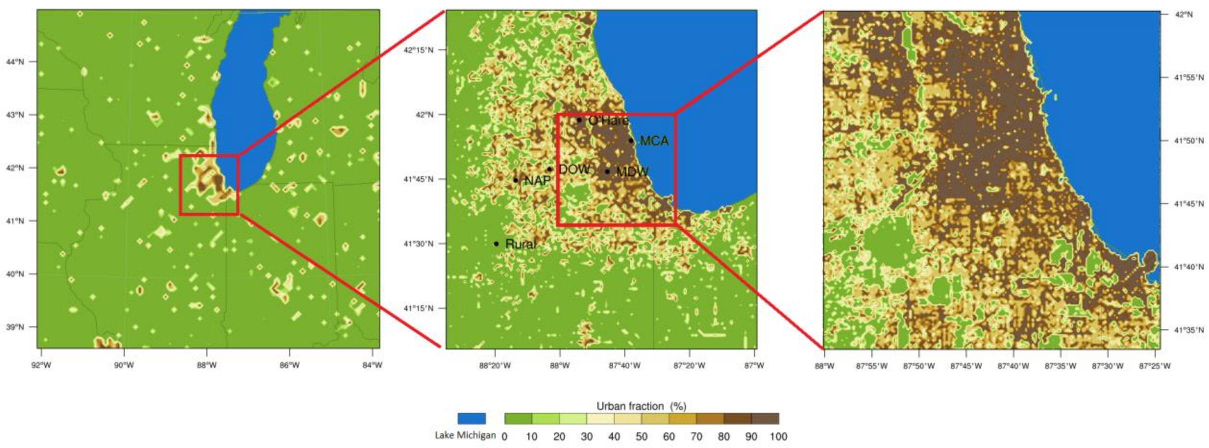

Figure S9. Urban fraction value in Chicago and surrounding area (domain d01 (left), d03 (middle) and d04 (right)). Black points in the middle panel are selected locations to represent the urban (urban fraction >80%)-suburban-rural (urban fraction <30%) area that corresponding the urban fraction.

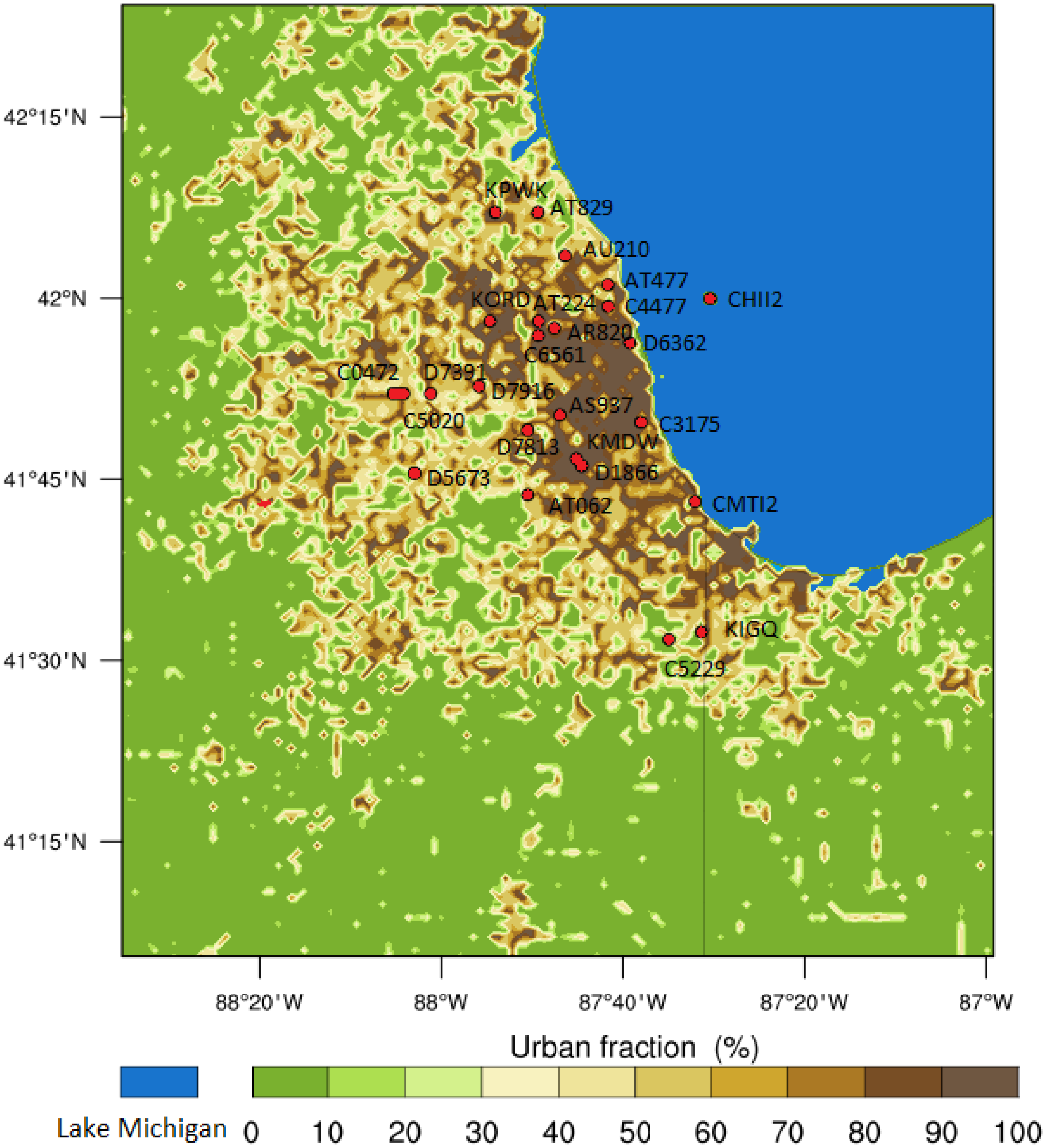

Figure S10. Locations and station names of 27 surface meteorology stations that provide data to MesoWest in urban Chicago. Legends are the same as Fig. S9 except the red dots represent station locations.

Figure S11 indicate the Hourly temperature, sensible heat flux and latent heat flux in urban and rural sites. The urban area has higher daytime sensible heat fluxes (~400 to 600 $W \cdot m^{-2}$) while the suburban and rural areas have ~200 $W \cdot m^{-2}$ sensible heat fluxes. It should be noted that the highest sensible heat flux occurs at MDW and O'Hare where the impervious surfaces are greater than 95%. On the contrary, the rural and suburban areas have notably higher latent heat fluxes (~350 $W \cdot m^{-2}$) during daytime while the latent heat fluxes are less than 150 W/m^2 in the urban area. Upward moisture flux (Figure S12) also has high values in the rural and suburban areas while lower in the urban region, especially at MDW. The rural area has a 3-4 times higher moisture flux than the urban area. High sensible heat flux and low latent heat flux resulted in the UHI effects in urban Chicago.

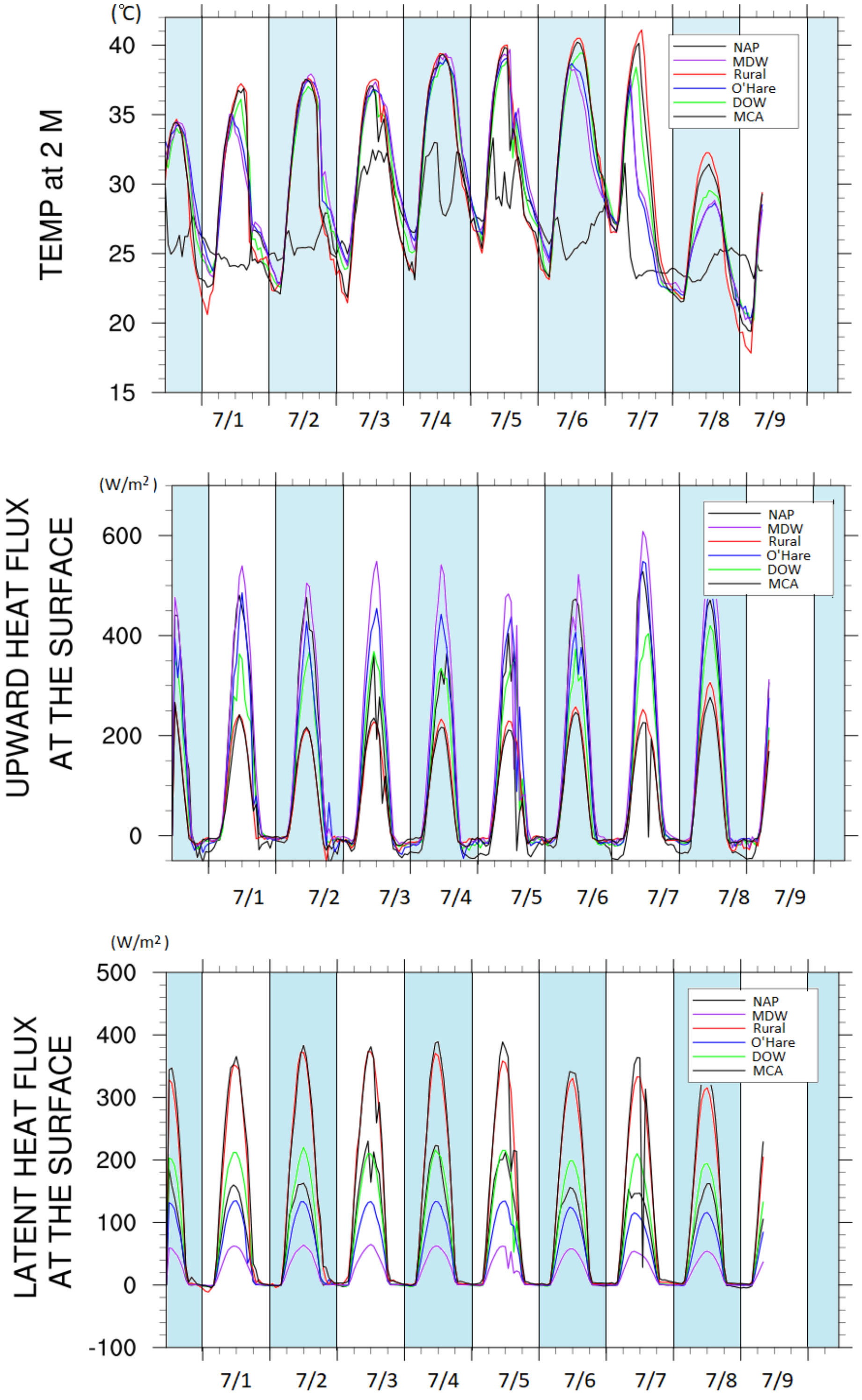

Figure S11. Hourly temperature (top), sensible heat flux (middle) and latent heat flux (bottom) variations for urban (MDW, MCA and O'Hare), suburban (NAP and DOW) and Rural areas.

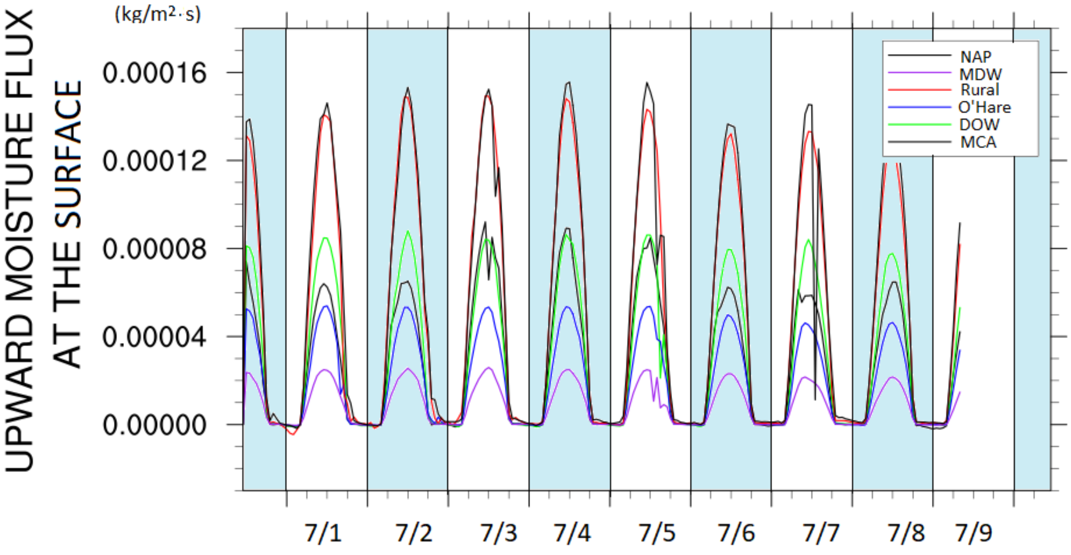

Figure S12. Upward moisture flux at 6 selected locations in d03.

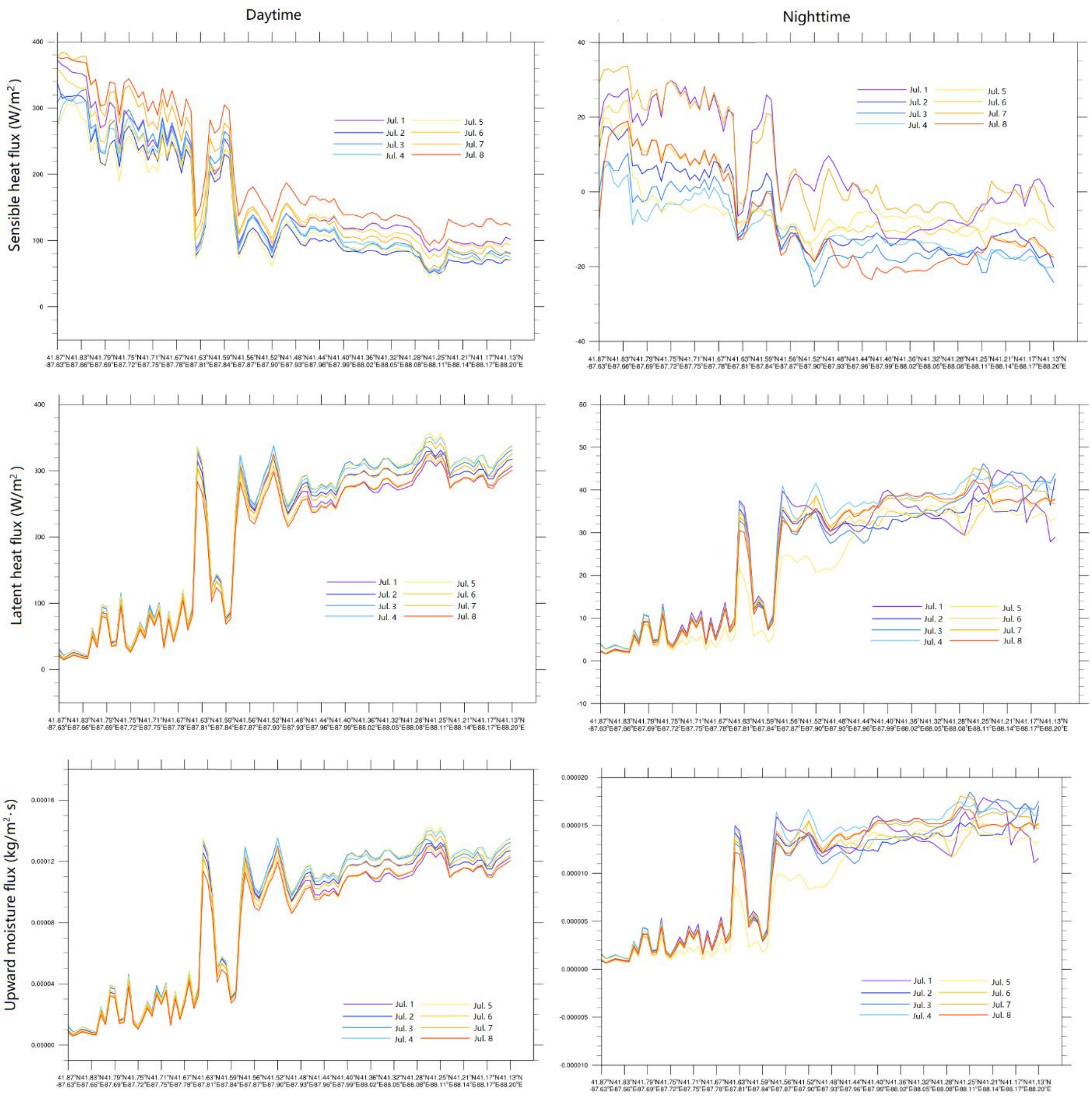

Figure S13. Daily variation of sensible heat flux, latent heat flux and upward moisture flux across the urban to rural trasncet shown in Figure 5 (panel A) during daytime (left) and nighttime (right).

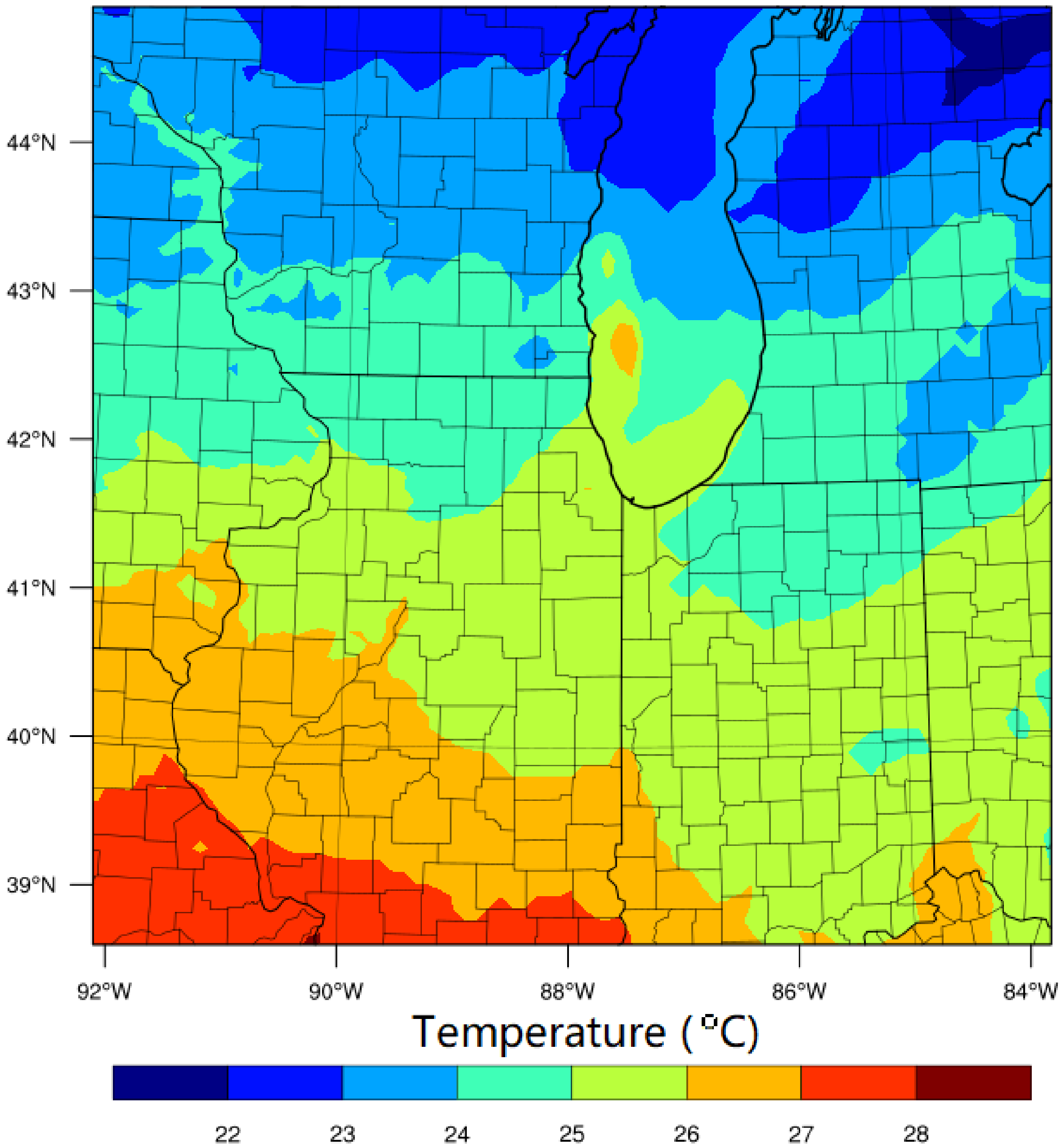

Figure S14. Climatological temperature (T95) during 2003-2012 for the d01. Data obtained from Daymet.

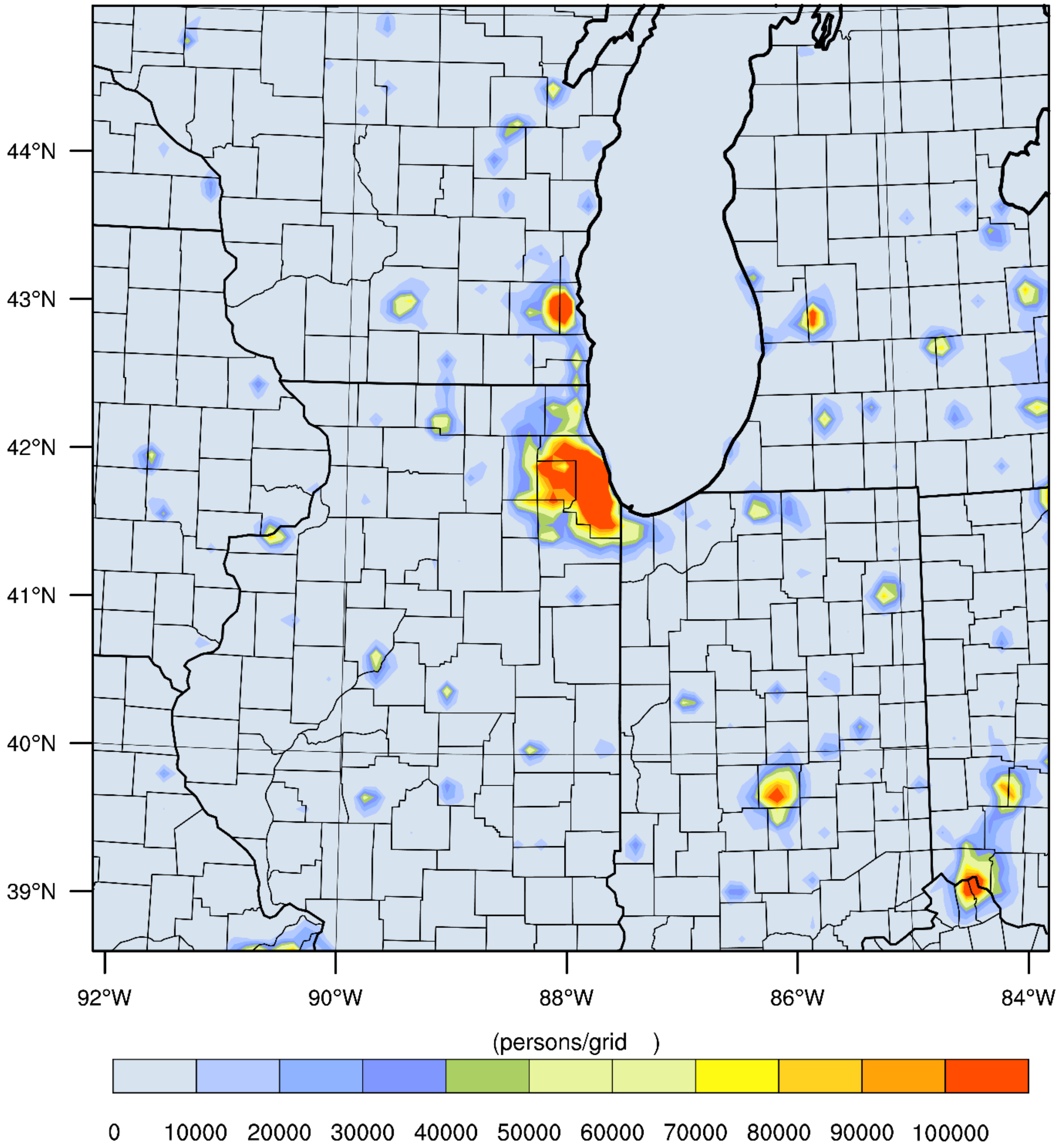

Figure S15. Population density based on data from 2015 for d01. Data obtained from Center for International Earth Science Information Network (CIESIN, https://sedac.ciesin.columbia.edu/data/set/gpw-v4-population-density-rev11).
